# Phenotypic and genome-based characterization of *Klebsiella* species from different One Health sources in South Africa reveals the presence of multidrug-resistant isolates

**DOI:** 10.3389/fmicb.2026.1752622

**Published:** 2026-05-29

**Authors:** Mohamed E. El Zowalaty, Bibek Lamichhane, Linda Falgenhauer, Dong-Yeop Lee, Emmanuel C. Eze, Sean G. Young, Asmaa Saleh, Stephen Forsythe, Oliver T. Zishiri, Dong-Hun Lee, Ji-Yeon Hyeon, Yosra A. Helmy

**Affiliations:** 1 School of Health Sciences, College of Health Sciences, University of KwaZulu-Natal, Durban, South Africa; 2 Department of Veterinary Science, Martin-Gatton College of Agriculture, Food and Environment, University of Kentucky, Lexington, KY, United States; 3 Hessian State Office for Health and Care, Department II.1 Health and Infection Protection, State Laboratory for Human Medicine, Dillenburg, Germany; 4 Department of Veterinary Medicine, College of Veterinary Medicine, Konkuk University, Seoul, Republic of Korea; 5 Department of Chemistry, Louisiana State University, Baton Rouge, LA, United States; 6 Department of Medical Laboratory Science, College of Health Sciences, Idaho State University, Pocatello, ID, United States; 7 Department of Health Data Science and Biostatistics, Peter O’Donnell Jr. School of Public Health, The University of Texas Southwestern Medical Center, Dallas, TX, United States; 8 Department of Pharmaceutical Sciences, College of Pharmacy, Princess Nourah bint Abdulrahman University, Riyadh, Saudi Arabia; 9 Foodmicrobe.com Ltd., Nottingham, United Kingdom; 10 Discipline of Genetics, School of Life Sciences, College of Agriculture, Engineering and Science, University of KwaZulu-Natal, Durban, South Africa

**Keywords:** antimicrobial resistance, *Klebsiella*, livestock, multidrug, One Health, prophage, whole genome sequencing

## Abstract

The emergence of antimicrobial-resistant and livestock-associated *Klebsiella* represents a serious public health concern, necessitating comprehensive genomic investigations. Bacterial strains were isolated from different domestic animals located in KwaZulu-Natal and Eastern Cape Provinces in South Africa. Antibiotic susceptibility testing was performed using the Kirby–Bauer disk diffusion method. Biofilm formation and hypermucoviscosity were investigated. Genomic DNA of the isolates was extracted, and whole-genome sequencing was performed using an Illumina MiSeq platform. Here, we report the isolation, molecular detection, virulence and antimicrobial resistance (AMR) determinants, as well as phylogenetic analyses of 16 *Klebsiella* strains using whole-genome sequence (WGS) analysis. Ten *Klebsiella pneumoniae* strains, three *Klebsiella variicola* and three *Klebsiella aerogenes* were detected. The assembled contigs were analyzed for AMR and virulence genes, K and O types, and multi-locus sequence type (MLST). To compare the sequenced strains to other previously sequenced *Klebsiella* species, a distance-based phylogeny method was used to confirm the taxonomic designation of the 16 *Klebsiella* genomes in an extended context of 169 genomes from the different *Klebsiella* species. Genomic analysis identified several antibiotic resistance genes conferring resistance to beta-lactams (*bla*_SHV-11-like_*, bla*_SHV-40/56/79/85/89-like_*, bla*_SHV-110/81-like_*, bla*_SHV-119_*, bla*_SHV-164/59-like_, and *bla*_LEN-16-like_), quinolones (*oqxA, oqxA-like, oqxB*, *oqxB*-like), tetracyclines [*tet(A)*], and fosfomycin (*fosA*-like). All isolates harbored genes conferring resistance to at least three antibiotic classes, thus classifying them as multidrug-resistant (MDR). Nine sequence types (ST-76, ST-128, ST-494, ST-617, ST-2465, ST-4039, ST-5470, ST-5478, and ST-5497) were identified using the MLST *K. pneumoniae* scheme in the *Klebsiella* collection. The latter three STs are being reported for the first time in the current study. MLST analysis revealed that the three *K. variicola* isolates belonged to ST-696 using the MLST *K. variicola* scheme. Although no hypervirulent strains were identified, the 16 *Klebsiella* isolates were *in vitro* biofilm producers and contained one to five intact prophages. The detection of MDR *K. pneumoniae* highlights the critical significance of genomic surveillance in monitoring the dissemination of antimicrobial resistance in livestock pathogens and within the food chain. The report of novel sequence types (ST-5470, ST-5478, and ST-5497) in *K. pneumoniae* present new allelic profiles, indicating the ongoing evolution and diversification within the species.

## Graphical abstract

**Figure:**
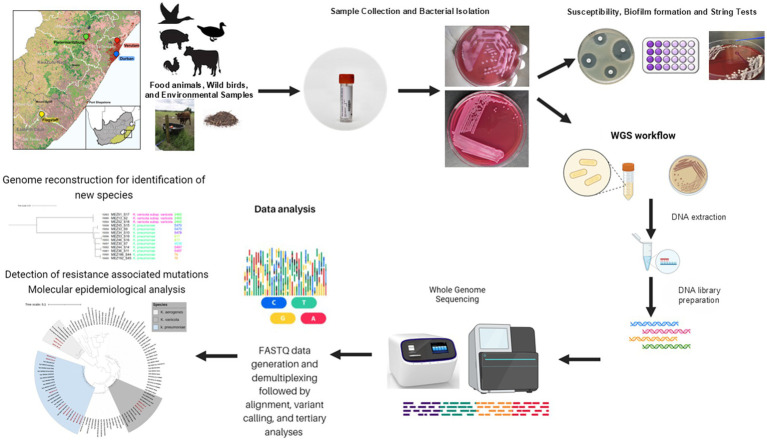

## 1 Introduction

Members of the genus *Klebsiella* are highly diverse Gram-negative bacteria, non-motile, usually capsulated, facultative anaerobic, lactose-fermenting, and opportunistic pathogens belonging to the family *Enterobacteriaceae*. They cause serious infections in humans and animals (Navon-Venezia et al., 2017). *Klebsiella* species are ubiquitous in humans, wild and domestic animals, plants, insects, and different environmental sources such as sewage, soil, and polluted water (Bagley, 1985; Dong et al., 2022). Currently, the genus *Klebsiella* includes 19 species and seven subspecies according to the List of Prokaryotic Names with Standing in Nomenclature (LPSN, https://lpsn.dsmz.de/genus/klebsiella accessed 8 May 2025) (Göker et al., 2025). *Klebsiella pneumoniae* (*K. pneumoniae*) is the most clinically significant *Klebsiella* species responsible for human infections. It is the most well-studied species and is listed in the 2024 WHO Bacterial Priority Pathogens List (BPPL) critical group pathogens by the World Health Organization (Sati et al., 2025; World Health Organization, 2024). *K. pneumoniae* is one of the highly multidrug-resistant (MDR) pathogens and is considered an important cause of life-threatening community-acquired pneumonia and difficult-to-treat infections (Tacconelli et al., 2018; Zhong et al., 2020; Wyres et al., 2020b; Kot and Witeska, 2024). *Klebsiella* species are one of the most important zoonotic enteric pathogens of epidemiologic concern and are responsible for morbidity and mortality in humans and livestock worldwide (Holt et al., 2015; Wyres and Holt, 2018). The high prevalence of third-generation cephalosporin, carbapenem, and colistin resistance in *K. pneumoniae* has been increasingly reported (Wyres and Holt, 2018; Mobasseri et al., 2019). The extensive and inappropriate use of antimicrobial agents as growth promoters and in treating diseases in animals have contributed to the emergence of extensively drug-resistant, MDR, and pan drug-resistant *K. pneumoniae* strains (Sonnevend et al., 2017; Yang et al., 2019). In addition, *K. pneumoniae* strains are circulating and several studies have increasingly reported strains exhibiting both virulence and AMR determinants (Lam et al., 2019). *Klebsiella* is responsible for a significant proportion of MDR intestinal and extra-intestinal infections in humans and food animals (Hiroi et al., 2012; He et al., 2017; Bidewell et al., 2018).

Virulent and MDR *Klebsiella* strains have been detected and isolated from domesticated animals including bovine, poultry, swine, horses, pets, and from wild animals as well as food products and environmental sources from different regions worldwide (Yang et al., 2019; Wareth and Neubauer, 2021; Kim et al., 2005; Kabir et al., 2024). On the other hand, *K. pneumoniae* has been newly suggested as a foodborne zoonotic pathogen as previously reported (Riley, 2020; Silva-Bea et al., 2024; Dela et al., 2023). The detection and worldwide spread of antimicrobial resistance genes (ARGs) including colistin resistance in clinical and livestock -associated isolates have spurred the genomic surveillance efforts in searching for ARGs in animals and food chain (Liu et al., 2016). Colistin resistance in *K. pneumoniae* has been reported from different regions as detailed elsewhere (Ah et al., 2014), including Europe, North America, South America, Asia and South Africa. Furthermore, MDR especially carbapenem-resistant *K. pneumoniae* strains have been reported globally as a critical public health threat (Lee et al., 2017).

Genome sequence analysis of *Klebsiella* is essential because of the microorganism’s potential impact on human and animal health. Whole-genome analysis is an ideal approach to detect genomic variations, and provides valuable information enabling us to understand virulence, pathogenesis, associated mechanisms of AMR, host specificity, and phylogenetic relationships. However, currently only scarce data concerning the genome sequence of *Klebsiella* spp. isolated from livestock in Africa are available. Recently, samples obtained from livestock production systems in KwaZulu-Natal Province in South Africa yielded *Bacillus licheniformis, Enterobacter kobei, Enterococcus faecalis, Enterococcus faecium, Enterococcus durans, Escherichia coli, Lelliottia nimipressuralis, Listeria innocua,* and *Salmonella enterica* as previously reported (El Zowalaty et al., 2023b; El Zowalaty et al., 2022b; El Zowalaty et al., 2019; El Zowalaty et al., 2022a; El Zowalaty et al., 2023a; El Zowalaty et al., 2024; El Zowalaty et al., 2020b; El Zowalaty et al., 2020a). Here, we report the phenotypic and genome-based characterization of sixteen *Klebsiella* strains comprising three species isolated from different One Health samples—livestock (goat, chicken, cow, sheep, and pigs), wildlife (wild duck), and environmental samples (water, wastewater, feed, and soil) in KwaZulu-Natal and Eastern Cape Provinces in South Africa.

## 2 Materials and methods

### 2.1 Sample collection

**Figure 1:**
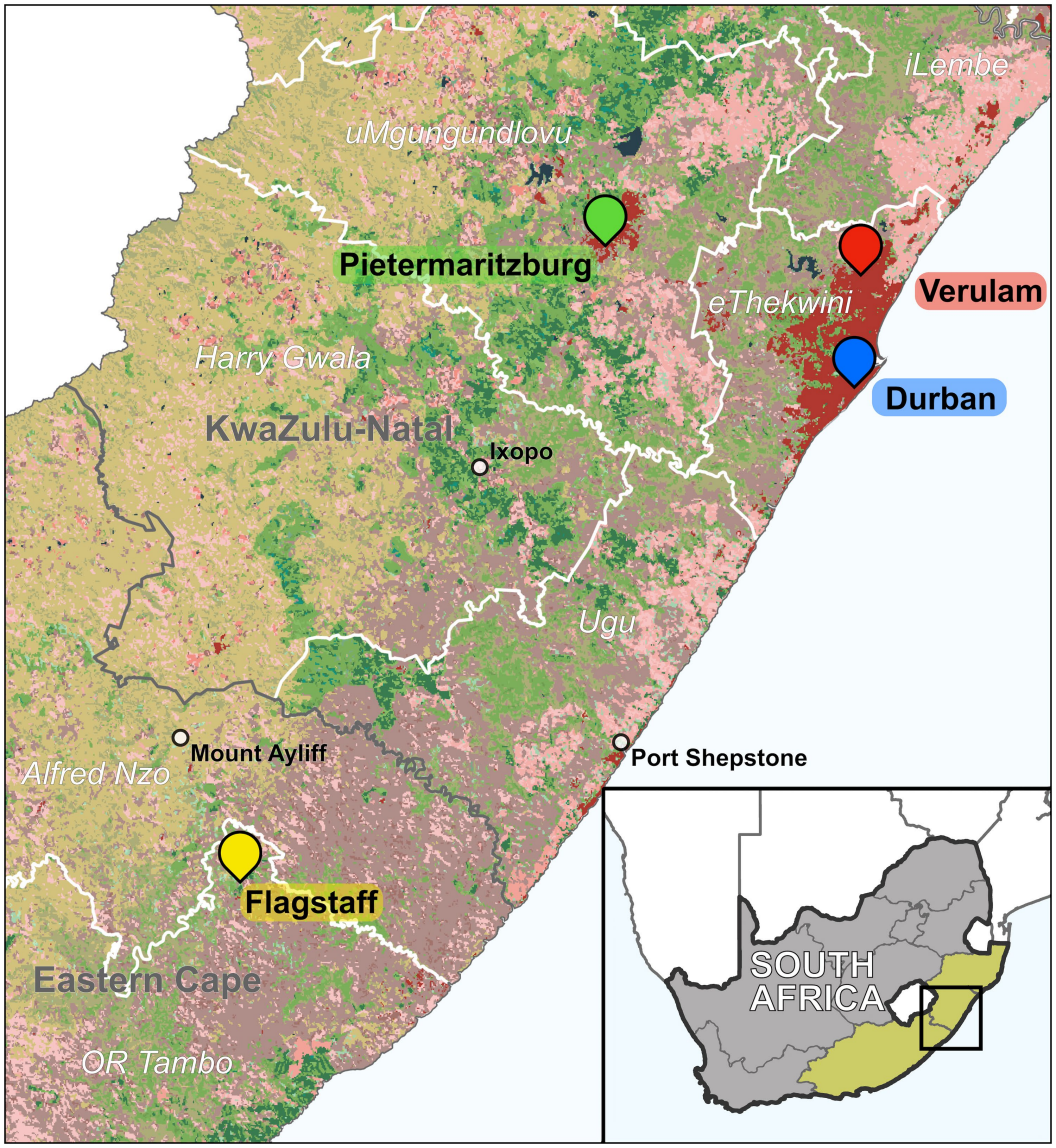
Sampling locations in the present study where the samples were collected from livestock farms in KwaZulu-Natal and Eastern Cape Provinces in South Africa. The four sampling locations are represented by blue pin (Durban), red pin (Verulam), green pin (Pietermaritzburg), and yellow pin (Flagstaff). The map was made using ArcGIS Pro [v3.1, Environmental Systems Research Institute (ESRI), Redlands, CA, USA].

One hundred and ninety-seven samples were collected from different animals housed in livestock farms, their environment, and farm workers in South Africa, as previously reported (El Zowalaty et al., 2023b). The samples were collected over a 9-month period between March and November in 2018. The livestock production farms were small-scale commercial farms located in Durban and Verulam (eThekwini Metropolitan Municipality), Pietermaritzburg (uMgungundlovu District Municipality) in KwaZulu-Natal Province, and Flagstaff (OR Tambo District Municipality) in Eastern Cape Province as shown in Figure 1. The de-identified human samples were collected from farm workers by swabbing the axillary area, groin, and hands. Samples were collected from animals by rectal, fecal, and oral swabbing using sterile cotton swabs. Fresh fecal samples from healthy animals, along with corresponding environmental samples from the farm settings (soil, feed, and water) were aseptically collected using sterile swabs following established protocols (Dweba et al., 2019; Mthembu et al., 2019). The swabs were immediately placed into screw capped sterile tubes containing 10 mL of 0.1% (*w*/*v*) peptone water (Merck, South Africa) and tubes were stored on ice while maintaining a cold chain temperature and immediately transported to the laboratory in the Discipline of Genetics, Westville Campus, the University of KwaZulu-Natal for further analysis.

### 2.2 Bacterial isolation and identification of *Klebsiella* species

A total of 1 mL peptone water sample was inoculated into 10 mL of Brain Heart Infusion broth (Merck, South Africa) for enrichment and incubated for a duration of 18–24 h at 37 °C. Bacterial cultures were streaked onto MacConkey agar (Merck, South Africa) and further incubated for a period of 18–24 h at 37 °C under aerobic conditions. Sixteen lactose-fermenting and mucoid colonies on MacConkey agar were purified and stocked for further analysis. The 16 presumptively identified *Klebsiella* isolates were transported to the University of Kentucky for further phenotypic characterization and the isolates were subsequently subjected to whole genome sequencing-based analysis as previously reported (El Zowalaty et al., 2023b).

### 2.3 Antimicrobial susceptibility testing

Antimicrobial susceptibility testing was performed against 14 different antibiotics using the Kirby-Bauer disc diffusion method and results were interpreted according to the standard breakpoints for inhibition zone provided by the Clinical and Laboratory Standard Institute and the European Committee on Antimicrobial Susceptibility Testing (EUCAST) as previously described (Clinical and Laboratory Standard Institute (CLSI), 2022; European Committee on Antimicrobial Susceptibility Testing (EUCAST), 2020). Briefly, the bacterial isolates were grown overnight in Mueller-Hinton (MH) growth medium (Fisher Scientific Inc., TX, USA) at 37 °C. The bacteria from overnight cultures were adjusted to an optical density at 600 nm (OD_600_) of 0.05 (2.5 × 10^7^ colony forming units per mL) in MH broth and 100 μL of the bacterial suspension (OD_600_ 0.05) was spread evenly on MH agar plates as described previously (European Committee on Antimicrobial Susceptibility Testing (EUCAST), 2021; Hailu et al., 2021). Antibiotic discs were purchased from Hardy Diagnostics, Santa Maria, CA, USA. Each isolate was tested for their performance against ampicillin (10 μg, AMP 10), amoxicillin-clavulanic acid (30 μg, AMC 30), cefotaxime (30 μg, CTX 30), ceftazidime (30 μg, CAZ 30), meropenem-vaborbactam (20/10 μg, MEV 30), chloramphenicol (30 μg, C 30), azithromycin (15 μg, AZM 15), gentamicin (10 μg, GM 10), nitrofurantoin (300 μg, F/M 300), tetracycline (30 μg, TE 30), ciprofloxacin (5 μg, CIP 5), levofloxacin (5 μg, LVX 5), fosfomycin (200 μg, FOS200), and tigecycline (15 μg, TGC15). The diameters of the zones of inhibition of the *Klebsiella* isolates were measured after incubating at 37 °C for 24 h under aerobic conditions as described previously (El Zowalaty et al., 2023b; Clinical and Laboratory Standard Institute (CLSI), 2022).

### 2.4 Broth microdilution test for colistin, imipenem and meropenem sensitivity

The antibiotic sensitivity for colistin sulfate (CT; AdipoGen Life Sciences, San Diego, CA, USA), imipenem monohydrate (IMP; AmBeed, Inc., Buffalo Grove, IL, USA), and meropenem trihydrate (MEM; TCI America, Portland, OR, USA) was determined using broth microdilution method for determining the minimum inhibitory concentration (MIC) as previously described by the Clinical and Laboratory Standard Institute (Clinical and Laboratory Standard Institute (CLSI), 2022). Briefly, 100 µL of overnight grown *Klebsiella* isolates (2.5 x 10^7^ CFU/mL) were incubated in the presence of 20 µL of each antibiotics (colistin sulphate (0.5–32 µg/mL), imipenem monohydrate (0.5–32 µg/ mL), and meropenem trihydrate (0.5–32 µg/mL)) diluted in HyClone™ HyPure molecular biology grade water (Cytiva, Marlborough, MA, USA) for 24 h at 37 °C under aerobic conditions. The growth of the antibiotics-treated isolates was compared with the untreated *Klebsiella* isolates (100 µL of each isolates + 20 µL of HyClone™ HyPure water) to determine the MIC. Similarly, 120 µL of sterile MH broth was also used as control. The results were interpreted as described by the CLSI guidelines (Clinical and Laboratory Standard Institute (CLSI), 2022). The MIC breakpoints (µg/mL) for colistin against *Klebsiella* spp. were interpreted according to the EUCAST guidelines. Resistance to colistin was defined as an MIC of ≥4 μg/ml (European Committee on Antimicrobial Susceptibility Testing (EUCAST), 2009).

### 2.5 Haemolysis test

Haemolytic phenotype was tested using sheep blood agar plates (Hardy Diagnostics, USA) containing 5% defibrinated sheep blood. Production of haemolysis was read after overnight incubation at 37 °C.

### 2.6 String test for hypermucoviscosity

The hypermucoviscosity phenotype of the isolates was determined using the semi-quantitative string test where an isolate was designated hypermucoviscous by the formation of viscous strings of at least 5 mm in length (Fang et al., 2004; Russo and Marr, 2019). The overnight-grown isolates were inoculated on sheep blood agar plates (Hardy Diagnostics, USA) and incubated at 37 °C for 24 h. An inoculation loop was then used to stretch out the colony to form a string on the plate and the string length was measured as previously described (Soto et al., 2020). Two independent experiments were performed.

### 2.7 Biofilm formation

Biofilm formation assay was performed as previously described (Danese et al., 2000; Kabir et al., 2025) with minor modifications. Briefly, the bacterial isolates were grown overnight in MH broth and adjusted to an OD_600_ of 0.05 (2.5 × 10^7^ CFU/mL). A volume of 200 μL of each isolate (OD_600_ 0.05) was added to a 96 well plate and incubated at 37 °C for 24 h under aerobic conditions without shaking. Two hundred microliters of sterile MH broth were used as a negative control. The bacteria were then removed, and the microtiter wells were washed with distilled water once and dried at room temperature. For detection of adherent biofilms, the wells were stained using 250 μL of 0.1% crystal violet (CV, Thermo Scientific, MA, USA) dissolved in Milli-Q® water and incubated at room temperature for 15 min. The crystal violet solution was then removed, and the wells were washed twice using distilled water to remove any remnant crystal violet solution. Following the removal of the crystal violet solution, the plate was air-dried and 250 μL of 30% glacial acetic acid (VWR Life Sciences, PA, USA) was added to each well to solubilize the CV-stained biofilms. The plate was incubated for 15–20 min, and the optical density at 550 nm (OD_550_) was measured (Bio-Tek, Winooski, VT, USA). Assays were performed three times, using duplicate wells in each independent assay. The absorbance of the negative control (MH medium) was used to normalize the results as previously described (O'toole, 2011). Two independent experiments with quadruple wells were performed.

### 2.8 Bacterial whole-genome sequencing

Whole-genome sequencing (WGS) of the bacterial isolates was performed at the University of Minnesota Genomic Center, Saint Paul, Minnesota, USA as previously reported (El Zowalaty et al., 2019; El Zowalaty et al., 2023b). Briefly, an aliquot of the bacterial culture in tryptic soy broth (Veterinary Diagnostic Laboratory, St. Paul, MN, USA) was diluted with 0.85% sterile saline solution (Veterinary Diagnostic Laboratory, St. Paul, MN, USA) to the desired inoculum density of 1 × 10^6^ CFU using Thermo Scientific™ Sensititre™ Nephelometer and the chilled culture tubes were submitted to the University of Minnesota Genomic Center for DNA extraction and sequencing. DNA extraction was performed using the Qiagen DNeasy Blood & Tissue kit (Lucigen, WI, USA) according to the manufacturer’s protocol. The DNA samples were quantified using a fluorimetric PicoGreen assay on a Synergy 2 plate reader (BioTech, Agilent, CA, USA). The purity of the samples was assessed via a Nanodrop 8000 instrument (Thermo Fischer, MA, USA). Sequencing libraries were prepared using the Nextera® XT DNA library preparation kit (Illumina Inc., CA, USA). Sequencing was performed using the Illumina MiSeq platform (Illumina Inc., CA, USA) using the v2 reagent kit, which yielded 250-bp paired-end reads.

### 2.9 Bioinformatics analyses

The 16 *Klebsiella* spp. draft genomes were generated in the Institute Pasteur (Paris, France) with an in-house developed workflow including the following read pre-processing steps: trimming and clipping with AlienTrimmer v.0.4.0 (Criscuolo and Brisse, 2013), sequencing error correction with Musket v.1.1 (Liu et al., 2013), coverage homogenization with khmer v.1.3 (Crusoe et al., 2015), assembly using SPAdes v3.14.1 (Bankevich et al., 2012), and annotation using PROKKA v.1.14.5. Read quality was checked and taxonomic classification of sequence reads data was performed based on Kraken2 (Wood et al., 2019).

Post-assembly processing steps using the assembled contigs files included several steps. The genomes were subjected to resistome, virulome, phylogenetic, and plasmid analyses as described below.

Kleborate (Lam et al., 2021) was used to detect sequences of concern and perform capsular typing including ICEKp-associated virulence loci [yersiniabactin (*ybt*), colibactin (*clb*), salmochelin (*iro*), hypermucoviscosity (*rmpA*)], virulence plasmid-associated loci [salmochelin (*iro*), aerobactin (*iuc*), hypermucoviscosity (*rmpA*, *rmpA2*)], antimicrobial resistance (acquired genes, mutation-based resistance, gene truncations and intrinsic *β*-lactamases) and K (capsule) and O antigen (LPS) serotype prediction (via *wzi* alleles and Kaptive). For *Klebsiella* species outside the *K. pneumoniae* species complex (*Kp*SC), Kleborate was used to accurately determine the species and report the presence of any accessory genes (AMR, virulence, K and O types).

In addition, Kleborate was used to analyze the *Kp*SC using the 7-loci multilocus sequence type (MLST) (Diancourt et al., 2005) and the 629-loci cgMLST (core genome multilocus sequence typing) (Bialek-Davenet et al., 2014) schemes. Analysis of MLST types of *K. aerogenes* and the three *K. pneumoniae* isolates that showed a new MLST type in the Kleborate analysis, was performed using PubMLST^1^ and Bigsdb,^2^ respectively. Analysis of MLST types of *K. variicola* was performed using the MLST typing scheme for *K. variicola*^3^ (Barrios-Camacho et al., 2019).

A more extensive analysis of AMR genes was performed using ResFinder-2.2 implemented in the CGE pipeline (Thomsen et al., 2016) including all *Enterobacterales* AMR genes. The classification of β-lactamase genes, including their extended-spectrum status, was determined using the Beta-Lactamase DataBase (BLDB) as previously reported (Naas et al., 2017). Plasmids were detected in the assembled contig files using the ABRicate software^4^ (version 1.0.1) by performing a BLAST search against the plasmid replicons included in the PlasmidFinder 2.1 database (Carattoli et al., 2014). The virulence genes of the isolates were analyzed using ABRicate against the virulence factor database (VFDB) (Chen et al., 2005), with a cut-off of minimum 80% sequence identity and 80% coverage. The presence of virulence genes/virulence determinants associated with hypervirulence (*rmpA*, *rmpA2*, *iucA*, *irp2*, K1/K2 capsule type) was additionally tested using the Nucleotide Basic Local Alignment Search Tool (BLASTN) and the sequence of PCR primers used in the study by Rodríguez-Medina et al. (2024).

A distance-based phylogeny method was used to confirm the taxonomic designation of the 16 *Klebsiella* genomes in an extended context of 169 genomes from the different *Klebsiella* species. The genomes used in this phylogenetic analysis included the *K. variicola* type strain F2R9 (Garza-Ramos et al., 2021), *K. aerogenes* type strain ATCC 13048,^5^ the *K. pneumoniae* type strain ATCC 13883,^6^ and the hypervirulent *K. pneumoniae* model strains [NTUH-K2044 (Wu et al., 2009), ATCC 43816 (Broberg Ca et al., 2014) and SGH10 (Wyres et al., 2018)]. In addition, complete or assembled genomes of *K. variicola* (*n* = 45), *K. aerogenes* (*n* = 12) and *K. pneumoniae* (*n* = 107) from animals and environment downloaded from the Bacterial and Viral Bioinformatics Resource Center (BV-BRC)^7^ were used to investigate the genetic relationships among the isolates (Supplementary Table 1). kSNP4 (Hall and Nisbet, 2023) was used to identify core genome single nucleotide polymorphisms (SNPs) with the “-core -ML” options. This method was used to identify core-genome SNPs without requiring alignment. FastTree (Price et al., 2010) was used to construct the SNP-based maximum likelihood (ML) tree, which was automatically applied in the kSNP pipeline. To compare and visualize features associated with AMR and virulence, we used a previously reported pan-drug resistant *K. pneumoniae* strain (*kp* Nevada) (Kaur et al., 2024) as a basis for comparative genomic analysis using cgView.^8^ This strain was chosen as it was expected to encode the majority of *Klebsiella* AMR determinants. Comparison of the *K. pneumoniae* plasmid (A) Pkp1-19 (Accession Number: CP012884.1) and (B) a *K. pneumoniae bla*_NDM-1_-encoding plasmid p2 (Accession number: CP009115.1) with MEZKP30, MEZKP33, MEZKP34, MEZKP38, MEZKP45, MEZKP48, MEZKP50, MEZKP186, and MEZKP192 were carried out using cgView server (see footnote 8).

### 2.10 Comparison of *Klebsiella* isolates from the present study with those from BIGSdb/PubMLST

Quality control and assembly of raw data was performed using the bioinformatic pipeline ASA^3^P (Schwengers et al., 2020). Isolates depicting the same ST as those reported in the present study were downloaded as FASTA sequences from Bacterial Isolate Genome Sequence Database (BIGSdb) (https://bigsdb.pasteur.fr/klebsiella/, for *K. pneumoniae* and *K. variicola*) or *Klebsiella* PubMLST and analyzed by sequence types to determine close phylogenetic relatives to the study isolates. Novel sequence types were assigned by BIGSdb curator. The phylogenetic analysis was performed using ParSNP implemented in the HARVEST Suite (Treangen et al., 2014). The phylogenetic trees produced in ParSNP were annotated using iTOL v.6.9 (Letunic and Bork, 2024). Trees were finalized using InkScape 0.9.1. Core genome SNPs were extracted from the ParSNP output and SNP counts were determined by MEGA 11 (Tamura et al., 2021).

### 2.11 Identification of prophages in *Klebsiella* strains

We investigated the prophage types integrated into the genome in each strain. Prophage sequences within the genomes of the 16 *Klebsiella* isolates were detected using the PHASTER (Phage Search Tool Enhanced Release; https://phaster.ca/) bioinformatics platform (Arndt et al., 2016). Only prophages classified as intact by PHASTER (score > 90) were indicated. The name of each prophage type was described using the most common phage indicated by the PHASTER database.

## 3 Results

### 3.1 Prevalence of *Klebsiella*

In the present study, we report a *One Health* and whole genome-based approach using WGS data for 16 *Klebsiella* isolates, recovered from environmental (water), livestock, and wild duck (oral and fecal) samples collected from South Africa as shown in Figure 1. Illustration of the sample collection, isolation, and WGS workflow is shown in the graphical abstract. A total of 197 non-duplicate samples were collected from healthy animals from livestock production systems, horses, wild ducks, and the farm environments, and samples were screened for the presence of *Klebsiella* using conventional microbiological methods. In the present study, a total of 182 samples were from animals (livestock, horses, and wild animals) including 141 samples collected from livestock (*n* = 135), horses (*n* = 2) and wild ducks (*n* = 4), and 41 samples were obtained from the surrounding environment (water, soil, and feed) as shown in Table 1. The 135 samples were obtained from different livestock included fecal (*n* = 85), oral (*n* = 50) swab samples, and the farm environment (water and soil) including 9 samples from soil (one sample from goat farm, one sample from sheep farm, two samples from chicken farm, and five samples from cow farm) and 7 samples from drinking water troughs (one sample from cow farm, three samples from chicken farm, and three samples from goat farm).

**Table 1:** Samples collected in the present study including the number and fecal, oral, environmental type of samples.

Host	Chicken	Pig	Goat	Duck domestic	Cow	Sheep	Horse	Wild duck	Soil	Water	Waste water	Feed	Humans	Total
Number/type	40 fecal	6 fecal	12 fecal	3 fecal	10 fecal	14 fecal	2 fecal	4 oral	13	9	18	1	15	
	15 oral	11 oral	13 oral		9 oral	2 oral								
Total	55	17	25	3	19	16	2	4	13	9	18	1	15	
	137 (livestock + horse)							4 wild animals	41 environmental				15	
	182 non-human (horse, livestock, wild animal, and environmental) samples												15	197

The livestock samples were collected from different animals and their environment (water and soil) and included domestic chickens (*n* = 55), domestic ducks (*n* = 3), cows (*n* = 19), goats (*n* = 25), sheep (*n* = 16), and pigs (*n* = 17) as shown in Table 1. The two (*n* = 2) samples collected from horses were fecal swabs and the four (*n* = 4) samples collected from wild ducks (dappling duck, *Anas platyrhynchos*) were oral swabs. The 25 environmental samples included waste water (*n* = 18) samples and miscellaneous (*n* = 7) samples from the farm environment where the livestock samples were collected. The seven environmental samples included water (*n* = 2), feedlot (*n* = 1), soil (*n* = 4) samples. Moreover, a total of 15 de-identified human samples were collected from farm workers.

Based on the phenotypic colony morphology, 16 (8.1%) isolates out of 197 tested samples were presumptively identified as *Klebsiella* spp. The species determination was performed using Kleborate that defines the species based on a MASH distance analysis towards a *Klebsiella* reference database. The species of the isolates confirmed using WGS-based analysis were *K. pneumoniae* (5%, *n* = 10, three from water, two from sheep, two from pig, one from chicken, one from wild duck, and one from a cow), *K. aerogenes* (1.5%, *n* = 3, two from pig samples and one from wild duck), and *K. variicola* (1.5%, *n* = 3, chicken). *K. pneumoniae* strains (*n* = 10) were isolated from samples collected from five different animal species including pigs (*n* = 2), cows (*n* = 1), chickens (*n* = 1), wild duck (*n* = 1), and sheep (*n* = 2), and environmental water samples (*n* = 3) as shown in Table 1. *Klebsiella* isolates were recovered from different animals using oral (*n* = 8) and fecal (*n* = 5) samples as shown in Table 2. Among the *Klebsiella* isolates (*n* = 16), the majority (*n* = 11 out of 197, 5.6%) of *Klebsiella* strains were isolated from samples collected from KwaZulu-Natal (KZN) Province while five (2.5%) isolates were isolated from samples collected from Eastern Cape (EC) Province. The swab samples obtained from the farm workers were negative when cultured using MacConkey agar and *Klebsiella* isolates were not recovered from these human swab samples. Culture of the swab samples collected from humans using mannitol salt agar revealed yellow colonies which were not further analyzed in this study.

**Table 2:** Sample types, host species and numbers of *Klebsiella aerogenes, Klebsiella pneumoniae* and *Klebsiella variicola* isolated from livestock production systems, wild birds and environmental water samples in the present study.

Host	Sample type	*Klebsiella* species	Number of isolates	Location (city, Province)	Isolate(s) ID
Chicken	Fecal	*Klebsiella variicola*	3	Verulam, KZN	MEZKV13, MEZKV51, MEZKV52
Chicken	Oral	*Klebsiella pneumoniae*	1	Verulam, KZN	MEZKP58
Duck (wild)	Oral	*Klebsiella aerogenes*	1	Durban, KZN	MEZKA31
		*Klebsiella pneumoniae*	1		MEZKP30
Cow	Oral	*Klebsiella pneumoniae*	1	Verulam, KZN	MEZKP38
Pig	Oral	*Klebsiella aerogenes*	2	Flagstaff, EC	MEZKA37, MEZKA43
		*Klebsiella pneumoniae*	2		MEZKP33, MEZKP45
Sheep	Fecal	*Klebsiella pneumoniae*	2	Verulam, KZN	MEZKP48, MEZKP50
Water	Water	*Klebsiella pneumoniae*	1	Flagstaff, EC	MEZKP34
Waste water	Water	*Klebsiella pneumoniae*	2	Pietermaritzburg, KZN	MEZKP186, MEZKP192

### 3.2 Antibiotic sensitivity profiling

**Figure 2:**
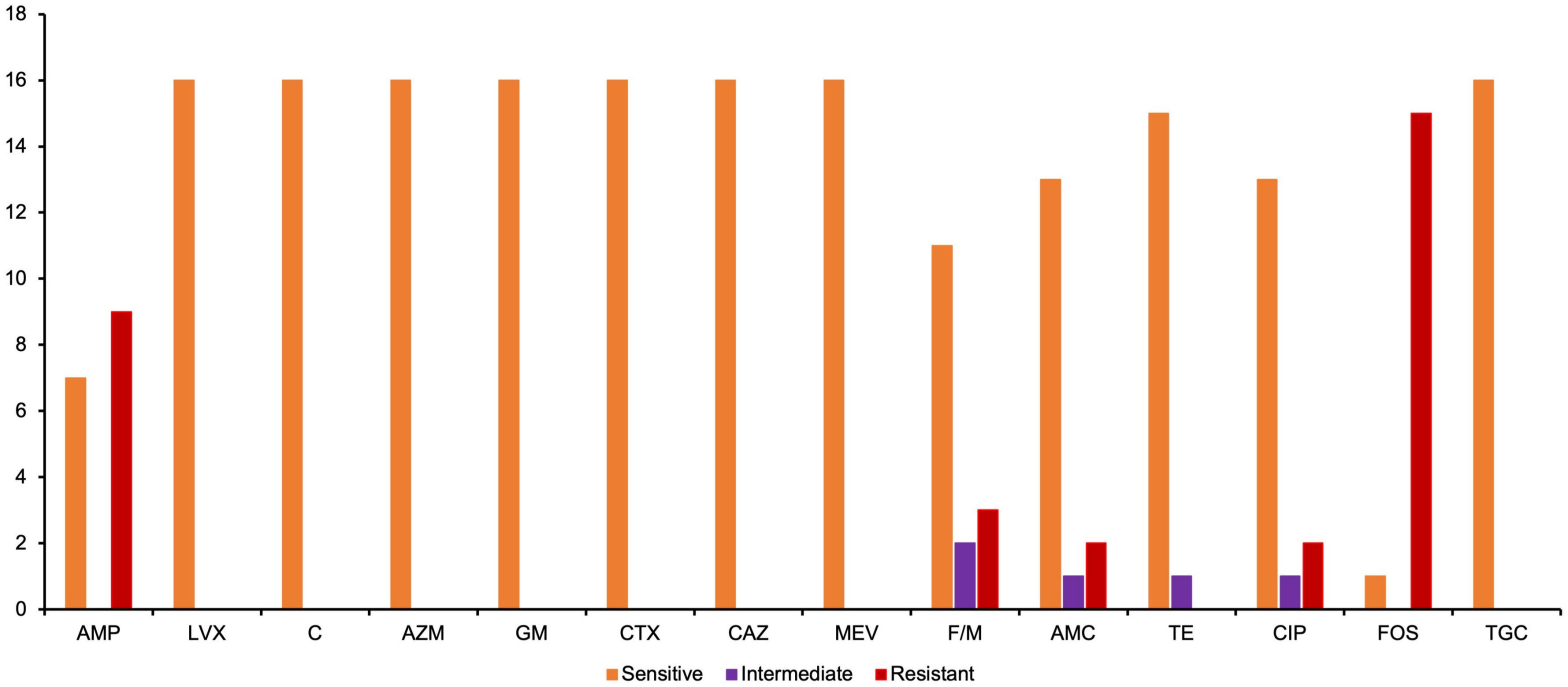
Antibiotic sensitivity patterns of *Klebsiella* isolates reported in the present study. The highest percentage of resistance was found against fosfomycin (93%) followed by ampicillin (56.25%), nitrofurantoin (18.75%, *n* = 3), amoxicillin-clavulanic acid (12.5%), and ciprofloxacin (12.5%). The numbers of sensitive, intermediate, and resistant isolates are shown on the Y-axis and the names of antibiotics are shown on the X-axis.

The results of the antimicrobial susceptibility testing of the 16 *Klebsiella* isolates revealed that all the isolates were resistant to at least three classes of the antibiotics tested. The highest level of resistance was against fosfomycin (93%, *n* = 15) followed by ampicillin (56.25%, *n* = 9), nitrofurantoin (18.75%, *n* = 3), amoxicillin-clavulanic acid (12.5%, *n* = 2), and ciprofloxacin (12.5%, *n* = 2). The 16 (100%) *Klebsiella* isolates were susceptible to levofloxacin, chloramphenicol, azithromycin, gentamicin, cefotaxime, ceftazidime, meropenem-vaborbactam, and tigecycline as shown in Figure 2. The 16 isolates were sensitive to tetracycline except one isolate (MEZKP30) which was interpreted as intermediate. Two *K. pneumonaie* isolates (MEZKP30 and MEZKP50) were categorized as intermediate susceptible to nitrofurantoin and another *K. pneumonaie* isolate (MEZKP186) was intermediate susceptible to ciprofloxacin. One *K. aerogenes* isolate (MEZKA31) showed intermediate susceptibility to amoxicillin-clavulanic acid.

Similarly, the broth microdilution method for MIC showed that the majority of the isolates (93.75%, *n* = 15) were resistant to colistin sulphate with MIC as high as 32 μg/mL. A single isolate (MEZKP33) demonstrated intermediate susceptibility to colistin (MIC= 2 μg/mL). All the 16 *Klebsiella* isolates were resistant to imipenem with MIC ≥8 μg/mL. One *K. aerogenes*, one *K. variicola* and two *K. pneumoniae* isolates were susceptible to meropenem. Five *K. pneumoniae*, one *K. aerogenes*, two *K. variicola* isolates were resistant to meropenem and imipenem. The MIC results of colistin, meropenem, and imipenem are shown in Table 3.

**Table 3:** MIC (μg/mL) for colistin, meropenem, and imipenem against *Klebsiella* isolates.

Isolate ID	Colistin	Meropenem	Imipenem
MEZKV13	32	8	8
MEZKV51	32	<0.5	8
MEZKV52	>32	8	16
MEZKA31	8	32	32
MEZKA37	8	<0.5	8
MEZKA43	16	2	8
MEZKP30	32	16	8
MEZKP33	2.4	4	8
MEZKP34	>32	2	8
MEZKP38	>32	2	8
MEZKP48	>32	<0.5	16
MEZKP45	32	16	16
MEZKP50	32	<0.5	16
MEZKP58	>32	16	16
MEZKP186	>32	64	8
MEZKP192	>32	2	8

Among the 16 *Klebsiella* isolates, the highest number of resistant isolates was observed in *K. pneumoniae* (62.5%, *n* = 10). This was followed by *K. aerogenes* and *K. variicola* (18.75%, *n* = 3 each). In the present study, the 16 *Klebsiella* isolates demonstrated MDR pattern as shown in Table 4. Multidrug resistance was defined as non-susceptibility to at least one agent in three or more classes (categories) of antibiotics as previously defined and reported (Magiorakos et al., 2012). Nine (56.3%) *K. pneumoniae* strains out of the 16 *Klebsiella* isolates were resistant to four different classes of antibiotics; carbapenems (imipenem and/or meropenem), fluoroquinolones (ciprofloxacin), phosphonic acids (fosfomycin) and polymyxins (colistin). One *K. pneumoniae* isolate (MEZKP30) was phenotypically resistant to three classes of antibiotics; imipenem and meropenem (carbapenems), fosfomycin (phosphonic acids) and colistin (polymyxins). It was categorized as “intermediate” according to the disk diffusion results for tetracycline. However, MEZKP30 was genotypically resistant to four different classes and the genome harbored different resistance genes; *tetA*, *bla*_SHV-110/81-like_, *oqxA*-like, *oqxB*-like, and *fosA*, *fosA7*-like. Two *K. aerogenes* strains were resistant to four classes of antibiotics: carbapenems (imipenem and/or meropenem), fluoroquinolones (ciprofloxacin), phosphonic acids (fosfomycin) and polymyxins (colistin). One isolate (MEZKA37) was resistant to three classes of antibiotics; carbapenems (imipenem), penicillin plus β-lactamase inhibitors (amoxicillin-clavulanic acid) and polymyxins (colistin). The three *K. variicola* strains were resistant to three classes of antibiotics; carbapenems (imipenem and/or meropenem), phosphonic acids (fosfomycin) and polymyxins (colistin). The phenotypic antibiotic resistance patterns of the 16 *Klebsiella* isolates are shown in Table 4.

**Table 4:** Phenotypic multidrug resistance profiles of *Klebsiella* species in the present study.

Isolate ID	Antibiotics	Number of antibiotic classes
MEZKV13	FOS, CT, IMP, MEM	3
MEZKV51	FOS, CT, IMP	3
MEZKV52	FOS, CT, IMP, MEM	3
MEZKA31	CIP, FOS, CT, IMP, MEM	4
MEZKA37	AMC, CT, IMP	3
MEZKA43	F/M, AMC, FOS, CT, IMP	4
MEZKP30	FOS, CT, IMP, MEM	3
MEZKP33	AMP, CIP, FOS, IMP, MEM	4
MEZKP34	AMP, FOS, CT, IMP	4
MEZKP38	AMP, FOS, CT, IMP	4
MEZKP48	AMP, FOS, CT, IMP	4
MEZKP45	AMP, FOS, CT, IMP, MEM	4
MEZKP50	AMP, FOS, CT, IMP	4
MEZKP58	AMP, FOS, CT, IMP, MEM	4
MEZKP186	AMP, F/M, FOS, CT, IMP, MEM	4
MEZKP192	AMP, F/M, FOS, CT, IMP	4

### 3.3 String test for hypermucoviscosity

**Figure 3:**
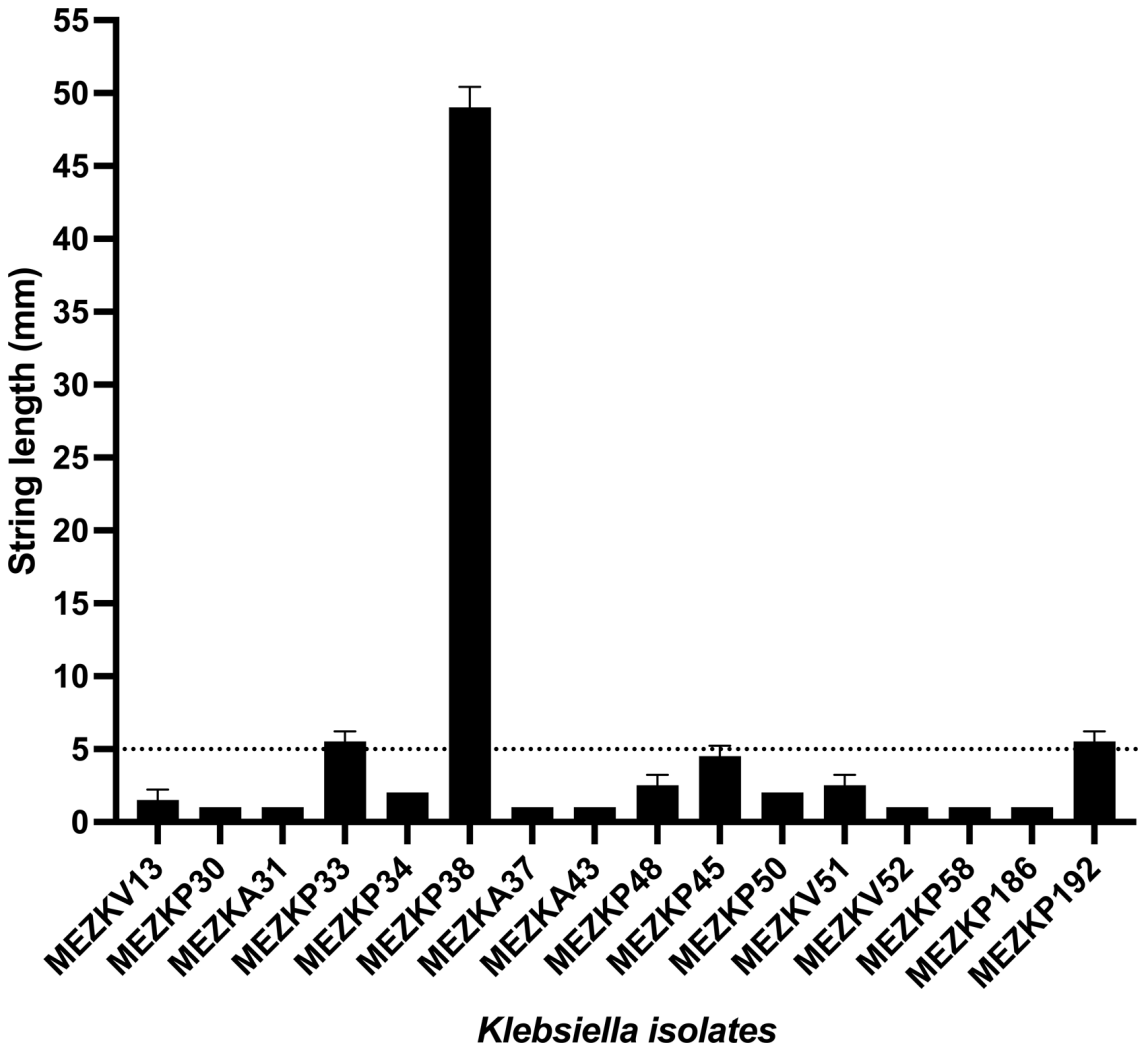
The length of strings formed by the *Klebsiella* isolates. The isolates with the string length more than 5 mm (represented by dotted line on *Y*-axis) were considered positive for string test. In the present study, three (MEZKP33, MEZKP38, and MEZKP192) isolates out of the 16 *Klebsiella* isolates were found to be positive for string test and had higher hypermucoviscosity as shown in the figure. MEZKP38 had the highest hypermucoviscosity with the string formation of 49 ± 1.41 mm.

All *K. aerogenes* and *K. variicola* isolates were negative for the string test. Three (18.7%) *K. pneumoniae* isolates out of the 16 *Klebsiella* isolates were positive for the string test and demonstrated higher hypermucoviscosity (HMV) as shown in Figure 3 and Supplementary Figure 1. One *K. pneumoniae* isolate (MEZKP38) exhibited the highest degree of hypermucoviscosity with the string length of 49 ± 1.41 mm on sheep blood agar as shown in Figure 3. Similarly, two additional *K. pneumoniae* isolates (MEZKP33 and MEZKP192) were also string-positive but displayed comparatively lower levels of hypermucoviscosity based on the length of the strings. The length of strings formed by the *Klebsiella* isolates is shown in Table 5. Notably, all the tested *Klebsiella* isolates were non-hemolytic when cultured on sheep blood agar.

**Table 5:** Measurement of viscous strings produced by *Klebsiella*.

Isolate ID	String length (mm ± SD)	Result
MEZKV13	1.5 ± 0.7	Negative
MEZKV51	2.5 ± 0.7	Negative
MEZKV52	1 ± 0	Negative
MEZKA31	1 ± 0	Negative
MEZKA37	1 ± 0	Negative
MEZKA43	1 ± 0	Negative
MEZKP30	1 ± 0	Negative
MEZKP33	5.5 ± 0.7	Positive
MEZKP34	2 ± 0	Negative
MEZKP38	49 ± 1.41	Positive
MEZKP45	4.5 ± 0.7	Negative
MEZKP48	2.5 ± 0.7	Negative
MEZKP50	2 ± 0	Negative
MEZKP58	1 ± 0	Negative
MEZKP186	1 ± 0	Negative
MEZKP192	5.5 ± 0.7	Positive

The bacteria producing strings more than 5 mm in length are considered positive for string test.

### 3.4 Biofilm formation

**Figure 4:**
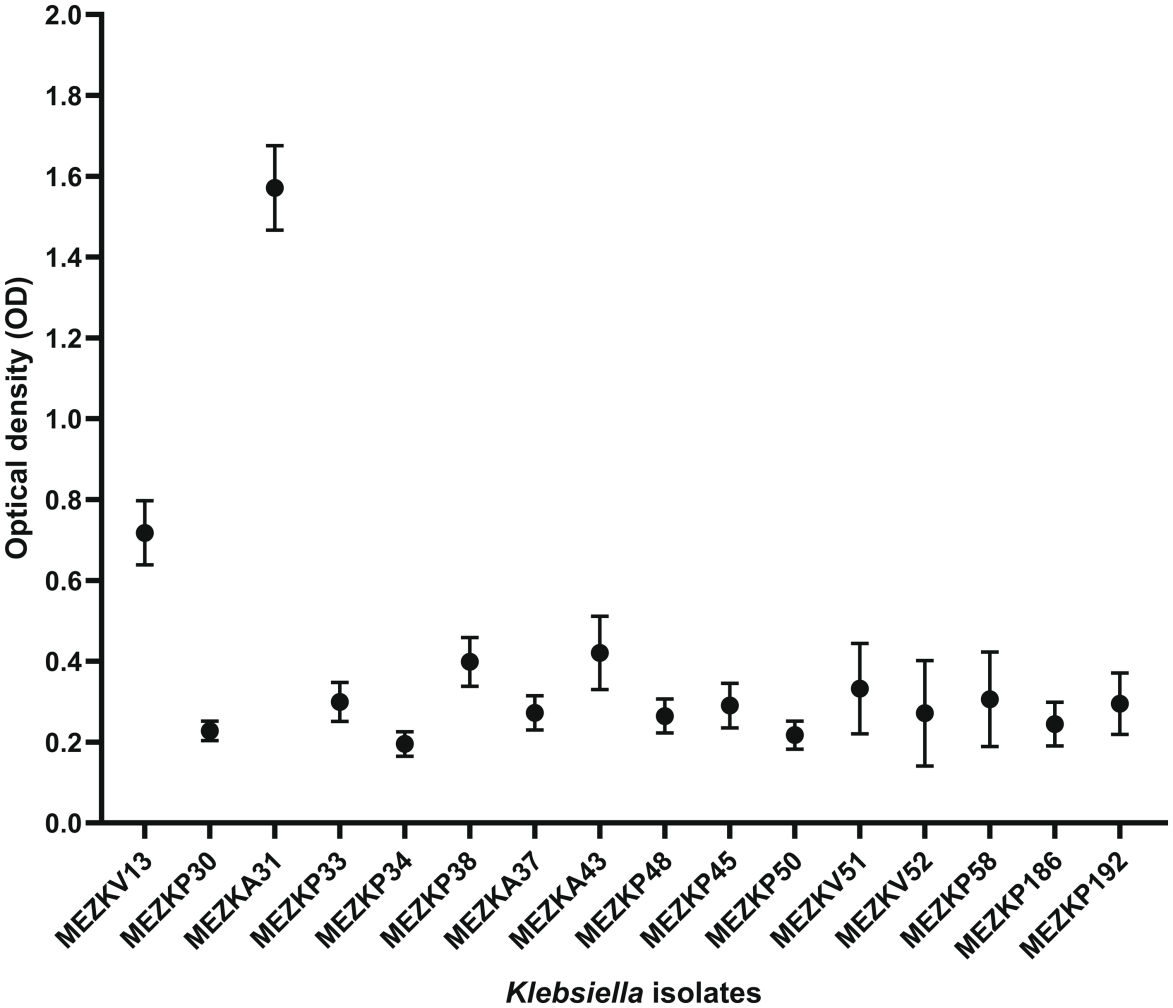
Biofilm formation of *Klebsiella* isolates. The 16 *Klebsiella* isolates demonstrated biofilm formation. The biofilm formation was found to be highest in isolate MEZKA31 followed by MEZKV13.

All 16 (100%) *Klebsiella* isolates demonstrated the formation of a biofilm *in vitro* as shown in Figure 4 and Table 6. The biofilm formation was found to be highest in *K. aerogenes* isolate MEZKA31 followed by *K. variicola* MEZKV13. Both HMV and non-HMV isolates were found to produce biofilms. Interestingly, the non-HMV isolates had higher biofilm forming capacity when compared to the HMV isolates.

**Table 6:** Average optical density/absorbance values (OD_550_) for the biofilm biomass produced by the 16 *Klebsiella* isolates.

Isolate ID	Average (OD_550_)	SD
MEZKV13	0.716	0.073
MEZKV51	0.327	0.100
MEZKV52	0.273	0.123
MEZKP30	0.230	0.025
MEZKA31	1.560	0.094
MEZKA37	0.272	0.038
MEZKA43	0.419	0.077
MEZKP33	0.302	0.043
MEZKP34	0.197	0.028
MEZKP38	0.397	0.048
MEZKP45	0.292	0.047
MEZKP48	0.266	0.043
MEZKP50	0.222	0.032
MEZKP58	0.311	0.108
MEZKP186	0.243	0.046
MEZKP192	0.300	0.070

### 3.5 Bacterial genomic sequence analysis using Kraken2

The Kraken2 analysis identified three different *Klebsiella* species including *K. pneumoniae* (*n* = 10), *K. variicola* (*n* = 3) and *K. aerogenes* (*n* = 3), with overall % of reads mapped ranging from 45.75% to 94.95% (Supplementary Table 2).

### 3.6 *De novo* assembly quality assessment and annotation

The overall genome assembly coverage of the 16 assemblies was >30 × (Supplementary Table 3). The number of contigs ranged from 65 to 297. The cumulative contig length was typical for that of *Klebsiella* species (5,066,070 bp—5,705,481 bp) with a minimum contig length >200 bp and a number of coding DNA sequences CDS ranging from 4,667 and 5,349 (see Supplementary Table 3). The average GC count accounted for 55.10% in *K. aerogenes* isolates, 57.27% in *K. pneumoniae* isolates and 57.53% in *K. variicola* isolates (see Supplementary Table 4).

### 3.7 Phylogeny-based taxonomy confirmation

A distance-based phylogeny method (Criscuolo, 2019) was initially used to confirm the taxonomic designation of the 16 *Klebsiella* genomes in an extended context of 92 genomes from the different *Klebsiella* species. The phylogenetic tree annotated with ST (accessible also at https://itol.embl.de/tree/157996425446811617297924) showed that the 16 *Klebsiella* genomes clustered consistently with the taxa identified by Kraken2 (Supplementary Figure 2).

### 3.8 Search for sequences of concern in *Klebsiella* genomic assemblies, *in silico* serotyping

The results of the genome assembly screen using Kleborate are depicted in Supplementary Table 5. Only *Klebsiella aerogenes* isolates harbored virulence genes, i.e., unknown Salmochelin variants.

In total, nine different K/O antigen combinations were detected in the 16 sequenced *Klebsiella* spp., each detected only in a certain multilocus sequence type.

*Klebsiella aerogenes* isolates harbored two different serotypes. KL68/O5 was detected in ST-128 isolates (*n* = 2) and KL42/O2 in the ST-494 isolate. *K. variicola* isolates harbored a single serotype (KL43/O3/O3a) that was also associated with a particular MLST-type (ST-2465 KP/ ST-696 KV).

*Klebsiella pneumoniae* isolates harbored six different serotypes, all of which were detected in a particular MLST type (KL122/O2, ST-617; KL125/O3b, ST-5470; KL24/O2, ST-5478; KL35/O5, ST-76; KL5/O3b, ST-4039; KL7/O2, ST-5497).

No plasmid incompatibility groups were detected in *K. aerogenes* and *K. variicola* isolates (Supplementary Table 4). In *K. pneumoniae* isolates, the number of plasmid incompatibility groups ranged between 0 and 12 (Supplementary Table 4). The most common plasmid incompatibility group were Col- and IncF-type plasmid incompatibility groups.

### 3.9 MLST determination

MLST results are detailed in Table 7 and Table 8. MLST analysis revealed that the three *K. variicola* isolates reported in the present study belonged to ST-2465 using the BIGSdb MLST *K. pneumoniae* scheme (Institut Pasteur, France) and to ST-696 using the MLST *K. variicola* (Instituto Nacional de Salud Pública, Mexico). Among the three *K. aerogenes* isolates, two belonged to ST-128, while MEZKA31 belonged to ST-494. The ten *K. pneumoniae* isolates were assigned to six STs including ST-76 (*n* = 2), ST-617 (*n* = 2), ST-4039 (*n* = 1), ST-5470 (*n* = 2), ST-5478 (*n* = 1), and ST-5497 (*n* = 2). Notably, the three latter STs represent previously unknown allele combinations and therefore were designated as novel STs.

**Table 7:** Allelic profiles of the novel *Klebsiella pneumoniae* STs reported in the present study.

Species	ST	*gapA*	*infB*	*mdh*	*pgi*	*phoE*	*rpoB*	*tonB*
*Klebsiella pneumoniae*	5470	10	1	1	1	9	5	24
*Klebsiella pneumoniae*	5478	2	1	2	1	1	4	23
*Klebsiella pneumoniae*	5497	2	3	2	1	1	4	24

**Table 8:** GenBank accession numbers, sequence types, and antibiotic resistance genes of *Klebsiella aerogenes, Klebsiella pneumoniae* and *Klebsiella variicola* genomes reported in the present study.

Isolate ID	Accession numbers		Species	MLST	Antibiotic resistance***			
	Sample	GenBank			Beta-lactam	Quinolone	Tetracycline	Fosfomycin
MEZKA31	SAMN18762342	GCA_020592275.1	*Klebsiella aerogenes*	ST-494	ND**	ND	ND	ND
MEZKA37	SAMN18762345	GCA_020592195.1	*Klebsiella aerogenes*	ST-128	ND	ND	ND	*fosA-like*
MEZKA43	SAMN18762347	GCA_020592115.1	*Klebsiella aerogenes*	ST-128	ND	ND	ND	*fosA-like*
MEZKP186	SAMN18762354	GCA_020591915.1	*Klebsiella pneumoniae*	ST-76	*bla*_SHV-164/59-like_^#^	*oqxA-like,oqxB-like*	ND	*fosA-like*
MEZKP192	SAMN18762355	GCA_020591985.1	*Klebsiella pneumoniae*	ST-76	*bla*_SHV-164/59-like_^#^	*oqxA-like,oqxB-like*	ND	*fosA-like*
MEZKP30	SAMN18762341	GCA_020592315.1	*Klebsiella pneumoniae*	ST-4039	*bla*_SHV-110/81-like_^#^	*oqxA-like,oqxB-like*	*tet(A)*	*fosA, fosA7-like (two fosA genes)*
MEZKP33	SAMN18762343	GCA_020592335.1	*Klebsiella pneumoniae*	ST-5470	*bla*_SHV-40/56/79/85/89-like_^#^	*oqxA-like,oqxB-like*	ND	*fosA5/−6-like (only one fosA gene)*
MEZKP34	SAMN18762344	GCA_020592215.1	*Klebsiella pneumoniae*	ST-5478	*bla*_SHV-11-like_	*oqxA-like,oqxB-like*	ND	*fosA-like*
MEZKP38	SAMN18762346	GCA_020592155.1	*Klebsiella pneumoniae*	ST-5497	*bla*_SHV-119_	*oqxA-like,oqxB-like*	ND	*fosA-like*
MEZKP45	SAMN18762348	GCA_020592085.1	*Klebsiella pneumoniae*	ST-5470	*bla*_SHV-40/56/79/85/89-like_^#^	*oqxA-like,oqxB-like*	ND	*fosA5/−6-like (only one fosA gene)*
MEZKP48	SAMN18762349	GCA_020592125.1	*Klebsiella pneumoniae*	ST-5497	*bla*_SHV-119_	*oqxA-like,oqxB-like*	ND	*fosA-like*
MEZKP50	SAMN18762350	GCA_020592075.1	*Klebsiella pneumoniae*	ST-617	*bla*_SHV-40/56/79/85/89-like_^#^	*oqxA,oqxB*	ND	*fosA6-like*
MEZKP58	SAMN18762353	GCA_020591975.1	*Klebsiella pneumoniae*	ST-617	*bla*_SHV-40/56/79/85/89-like_^#^	*oqxA,oqxB*	ND	*fosA6-like*
MEZKV13*	SAMN18762340	GCA_020592375.1	*Klebsiella variicola*	ST-696 (KV scheme), ST-2465 (KP scheme)	*bla*_LEN-16-like_	*oqxA-like,oqxB-like*	ND	*fosA-like*
MEZKV51*	SAMN18762351	GCA_020592025.1	*Klebsiella variicola*	ST-696 (KV scheme), ST-2465 (KP scheme)	*bla*_LEN-16-like_	*oqxA-like,oqxB-like*	ND	*fosA-like*
MEZKV52*	SAMN18762352	GCA_020592015.1	*Klebsiella variicola*	ST-696 (KV scheme), ST-2465 (KP scheme)	*bla*_LEN-16-like_	*oqxA-like,oqxB-like*	ND	*fosA-like*

*MEZKV13, MEZKV51, and MEZKV52 belonged to ST-2465 using the MLST *K. pneumoniae* scheme.

**ND; not detected.

***Antibiotic resistance genes were detected *in silico* using whole genome sequencing-based analysis using bioinformatics tools.

#Please note, that Resfinder gave more than one *bla*_SHV_—hit at an equal genome position and with identical identity/coverage scores, thus only one *bla*_SHV_ gene is present (no perfect hit).

MLST results for *K. pneumoniae* and *K. variicola* are also available in BIGSdb-Pasteur using this link, https://bigsdb.pasteur.fr/cgi-bin/bigsdb/bigsdb.pl?db=pubmlst_klebsiella_isolates&page=query&project_list=46&submit=1.

### 3.10 cgMLST-based analysis and phylogeny

The cgMLST results for *K. pneumoniae* and *K. variicola* are depicted available in BIGSdb-Pasteur using this link, https://bigsdb.pasteur.fr/cgi-bin/bigsdb/bigsdb.pl?db=pubmlst_klebsiella_isolates&page=query&project_list=46&submit=1. The 13 genomes of *Kp*SC species complex were compared based on the loci assigned by the cgMLST analysis in BIGSdb-Pasteur. The Neighbour-joining based phylogenetic tree was calculated from concatenated sequence alignments of 629 core loci. The tree (Supplementary Figure 3) is visualized on iTOL, and can be viewed using the link, https://itol.embl.de/tree/1579964253460351617320309

### 3.11 Analysis of antibiotic resistance and virulence genes

The results of the analysis of antibiotic resistance genes performed by Kleborate and ResFinder are depicted in Supplementary Tables 5, 6. Notably, none of the *Klebsiella* spp. isolates analyzed in the present study carried any genetic determinants associated with extended-spectrum β-lactamases (ESBLs) or carbapenemases.

None of the *K. aerogenes* isolates carried any β-lactam resistance genes (Supplementary Table 6). Two *K. aerogenes* isolates (MEZKA37 and MEZKA43) carried a *fosA*-like gene, while the remaining isolate (MEZKA31) did not harbor any fosfomycin resistance genes. All *K. aerogenes* isolates carried specific point mutations in the *ompK36* gene (N49S, L191Q, F207W, A217S, and D224E, Supplementary Table 6).

In the present study, 13 out of the 16 *Klebsiella* spp. isolates harbored genes encoding resistance to β-lactams, including *bla*_SHV-like_ (ten *K. pneumoniae* isolates) and *bla*_LEN-like_ genes (three *K. variicola* isolates).

All *K. variicola* isolates exhibited an identical profile of antimicrobial resistance genes and chromosomal point mutations (Supplementary Table 6). *K. variicola* isolates carried the β-lactam resistance gene (*bla*_LEN-16-like_), the fosfomycin resistance gene (*fosA*-like), and the quinolone resistance genes (*oqxA*-like and *oqxB*-like). Furthermore, all *K. variicola* isolates carried specific point mutations in the *acrR* (P161R, G164A, F172S, R173G, L195V, F197I, and K201M), *ompK36* (N49S, L59V, L191Q, F198Y, A217S, N218H, Q227N, L229V, and N304E), and *ompK37* (I70M and I128M) genes.

All *K. pneumoniae* strains carried at least one *bla*_SHV_ variant, all of which are classified as broad-spectrum β-lactamases (Table 8 and Supplementary Table 6). All *K. pneumoniae* isolates carried *fosA*-like genes encoding resistance to fosfomycin and *oqxA/oqxB*(-like) genes encoding resistance to quinolones. The particular fosfomycin resistance genes detected were *fosA* or *fosA*-like (*n* = 6), *fosA5*-like (*n* = 2), *fosA6*-like (*n* = 4) or *fosA7*-like (n = 1) genes. Only one *K. pneumoniae* isolate (MEZKP30) harbored *tetA* which encodes resistance to tetracyclines. All *K. pneumoniae* isolates carried specific point mutations in the *acrR* (P161R, G164A, F172S, R173G, L195V, F197I, and K201M), *ompK36* (N49S, L59V), and *ompK37* (I70M and I128M) genes.

The virulence gene profiles of the 16 isolates determined using ABRicate against VFDB are presented in Supplementary Table 7. The analysis revealed two distinct virulence gene profiles among the isolates. *K. pneumoniae* and *K. variicola* isolates carried virulence genes associated with adherence (*ompA, yagV-yagZ,* and *ykgK*) and enterobactin (*entA, entB,* and *fepC*). The *K. aerogenes* isolates possessed genes associated with adherence (*ompA, yagW*, and *yagZ*), enterobactin (*entA* and *entB*), flagella (*flgH, fliG, fliM,* and *fliN*), and salmochelin (*iroB* and *iroN*).

Only one virulence determinant was present in all *Klebsiella* spp. isolates from our study—*acrAB*. This virulence determinant is involved in resistance towards host-derived antimicrobial peptides. *K. aerogenes* isolates reported in the present study did not harbor any other virulence determinants.

*Klebsiella variicola* isolates harbored between three to four virulence determinants in addition to *acrAB*. Type I and type III fimbriae, and enterobactin were common to all *K. variicola* from our study. Type I fimbriae are involved in adhesion to surfaces, while type III fimbriae are crucial for biofilm formation. Enterobactin is a siderophore. Two out of three *K. variicola* isolates displayed the *RcsAB* virulence determinant involved in regulation of the capsule synthesis.

All *K. pneumoniae* isolates additionally harbored *acrAB* Type I, Type III fimbriae and *RcsAB*. One *K. pneumoniae* isolate (MEZKP48) harbored the LPS virulence determinant involved in immune modulation.

Using Kleborate and BLASTN of primers targeting gene sequences characteristic for hypervirulence, none of the genes or determinants characteristic for hypervirulence (*rmpA*, *rmpA2*, *iucA*, *irp2*, K1 or K2 capsule type) were detected in the genomes reported in the present study.

### 3.12 Plasmid replicons

No plasmid incompatibility groups were detected in *K. aerogenes* and *K. variicola* isolates (Supplementary Table 8). In *K. pneumoniae* isolates, the number of plasmid incompatibility groups ranged between 0 and 12 (Supplementary Table 8). The most common plasmid incompatibility groups were Col- and IncF-type plasmid incompatibility groups.

Several copies (two or five) of the Col(pHAD28) plasmid incompatibility group were detected in four isolates (MEZKP186, MEZKP192, MEZKP38, and MEZKP48). Furthermore, MEZKP38 and MEZKP48 harbored Col440II plasmid incompatibility groups. The IncR incompatibility group was detected twice in MEZKP186 and MEZKP192. Two different variants of IncFII incompatibility groups were detected—IncFII(pAR0022) (in MEZKP186, MEZKP34) and IncFII(pKP91) (in MEZKP186, MEZKP192, MEZKP34, MEZKP38, and MEZKP48). As shown, MEZKP186 harbored both IncFII variants and one even in two copies [IncFII(pKP91)]. Four different IncFIB variants were detected—IncFIB(pQil) (in MEZKP186, MEZKP192), IncFIB(K) (in MEZKP186, MEZKP192, MEZKP38, and MEZKP48), IncFIB(K)(pCAV1099-114) (in MEZKP38, MEZKP48) and IncFIB(pNDM-Mar) (in MEZKP30). Altogether, four out of five isolates harboring IncFIB plasmid incompatibility variants harbored two different variants of these. One IncFIA variant was detected [IncFIA(HI1)] present in four different isolates (MEZKP186, MEZKP34, MEZKP38, and MEZKP48).

**Figure 5:**
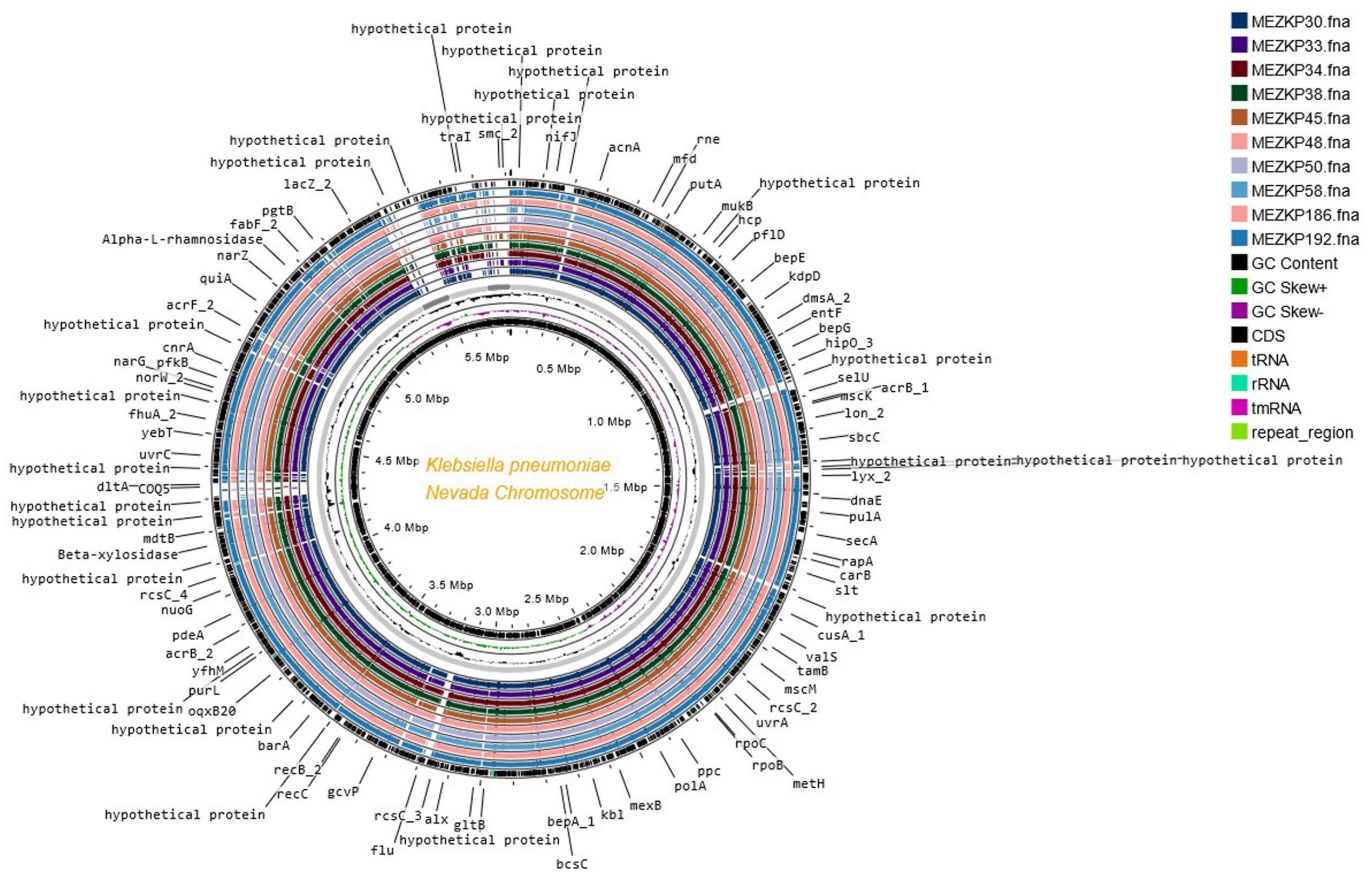
Comparison of *K. pneumoniae* Nevada chromosome (CP022125.1) with the draft genome of *K. pneumoniae* strains MEZKP30, MEZKP33, MEZKP34, MEZKP38, MEZKP45, MEZKP48, MEZKP50, MEZKP186, and MEZKP192 using GCview. The outer ring displays the CDS of the *K. pneumoniae* Nevada chromosome (Accession number: CP022125.1). The ten other rings show the BLAST results when the genome of *K. pneumoniae* strains MEZKP30, MEZKP33, MEZKP34, MEZKP38, MEZKP45, MEZKP48, MEZKP50, MEZKP186, and MEZKP192 are compared with the genome of *K. pneumoniae* Nevada chromosome with the hits (shaded region) indicating areas of homology. The inner rings (black) show the GC content while the pink and green show the GC skew positive and GC skew negative, respectively.

To further understand the resistance and virulence determinants present in the 10 *K. pneumoniae* genomes, a comparative chromosomal analysis was performed with a recently isolated pan-drug resistant *K. pneumoniae* strain. This comparison revealed multiple regions of sequence homology indicating the shared presence of numerous antimicrobial resistance and virulence-associated genes across both the pan-drug resistant *K. pneumoniae* strain and study genomes. Alignment of the contigs of the ten *K. pneumoniae* from this study to *kp* Nevada showed areas of multiple hits corresponding to the reference strain (Figure 5). However, there are few regions of difference (ROD) between the study strains and the reference (Figure 5). Comparative plasmid analysis with the two prominent *K. pneumoniae* plasmids, p2 and Pkp1-19 indicated that all the strains carried one or more plasmids (Supplementary Figures 4A,B).

### 3.13 Phylogenetic analysis

**Figure 6:**
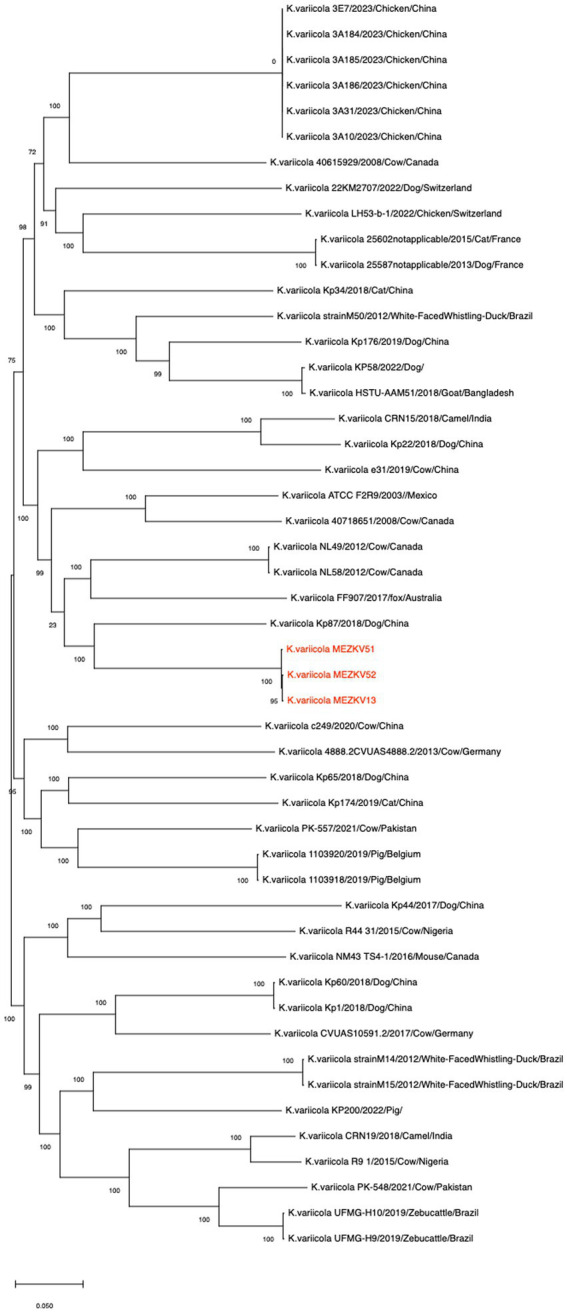
Maximum likelihood phylogenetic tree of 49 *Klebsiella variicola* genomes, including 45 genomes from BV-BRC and 3 isolates from this study. Core genome SNPs were identified using kSNP4, and the ML tree was constructed using FastTree.

**Figure 7:**
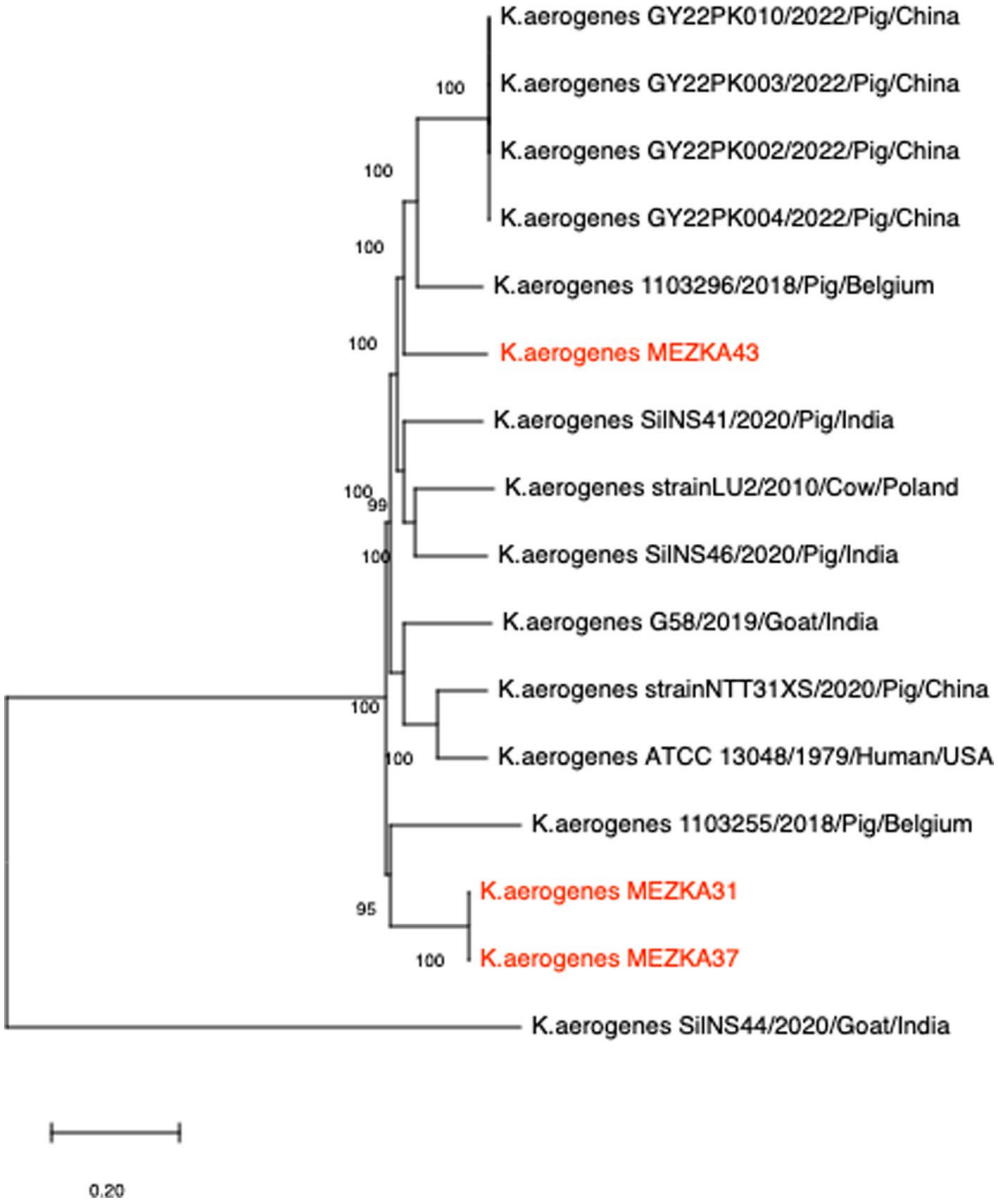
Maximum likelihood phylogenetic tree of 16 *Klebsiella aerogenes* genomes, including 12 genomes from BV-BRC and 3 isolates from this study. Core genome SNPs were identified using kSNP4, and the ML tree was constructed using FastTree.

**Figure 8:**
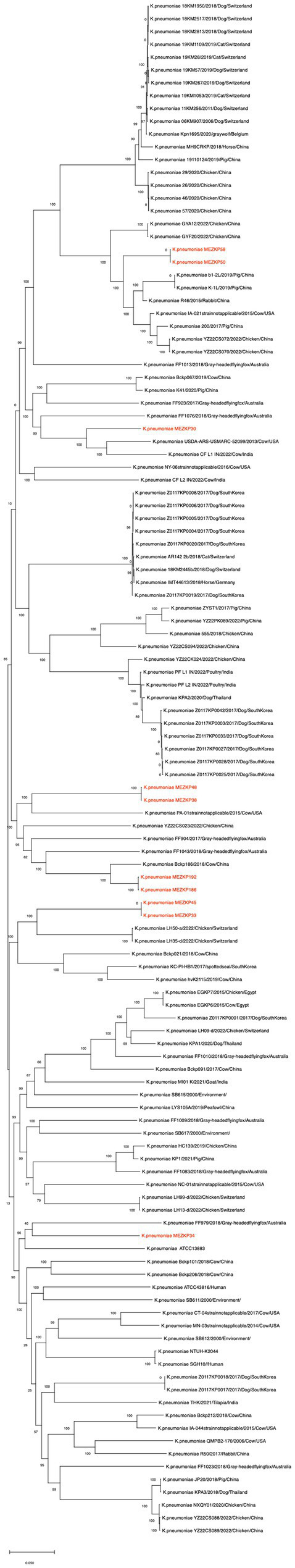
Maximum likelihood phylogenetic tree of 120 *Klebsiella pneumoniae* genomes, including 110 genomes from BV-BRC and 10 isolates from this study. Core genome SNPs were identified using kSNP4, and the ML tree was constructed using FastTree.

The genome sequences of *K. variicola* (*n* = 49), *K. aerogenes* (*n* = 16) and *K. pneumoniae* (*n* = 120) including the 16 genomes generated from the isolates of this study were analyzed using core genome SNP–based phylogenetic analysis to investigate their genetic relationships. The phylogenetic trees of the three different species are shown in Figures 6–8. Due to the limited availability of animal-derived genome sequences, broader comparative analysis was constrained; nonetheless, the isolates generally clustered according to their MLST profiles.

In the phylogenetic analysis of *K. variicola*, the three isolates from this study (ST-696) clustered closely together (Figure 6). For *K. aerogenes*, phylogenetic analysis of 15 genomes showed that two isolates from this study (MEZKA31 and MEZKA37) exhibited high similarity and clustered with a *K. aerogenes* isolate from a pig in Belgium. In contrast, MEZKA43 clustered with *K. aerogenes* isolates obtained from pigs in China and Belgium (Figure 7). The *K. pneumoniae* isolates sequenced in this study clustered into five groups according to their MLST profiles (Figure 8). Notably, none of the isolates from this study clustered with the hypervirulent *K. pneumoniae* model strains (NTUH-K2044 and SGH10), likely reflecting their assignment to different MLST types.

*Klebsiella pneumoniae* isolates sequenced in this study clustered according to the MLST results (Table 8 and Supplementary Table 9) demonstrating high genetic similarity among isolates with the same MLST (Figure 8). MEZKP33 and MEZKP45 (ST-5470) showed no SNPs (Supplementary Table 10), and MEZKP38 and 48 (ST-5497) showed 15 SNPs. MEZKP186 and MEZKP192 (ST 76) showed two SNPs, and MEZKP50 and MEZKP58 (ST-617) showed no SNPs. Notably, tables 3 and 4 showed that zero SNPs does not mean identical isolates. The isolates of this study were grouped, though distant, with the isolates from other countries (Figure 8). MEZKP33 and MEZKP45 isolates were grouped with two isolates from chickens in Switzerland; 5,889 and 5,894 SNP differences. The MEZKP186 and MEZKP192 were grouped with an isolate from a cow in the China showing 1,988 SNP differences.

### 3.14 Comparative analysis with isolates from BIGSdb/PubMLST

All ST-76 (*K. pneumoniae*, *n* = 82), ST-128 (*K. aerogenes*, *n* = 1), ST-494 (*K. pneumoniae*, *n* = 1), ST-617 (*K. pneumoniae*, *n* = 9), ST-2465 (*K. pneumoniae*, *n* = 1), and ST-4039 (*n* = 2) *K. pneumoniae/K. aerogenes* genomic sequences available in BIGSdb/PubMLST (all but ST-76 as of 30th of May 2024) were downloaded and analyzed by sequence types to determine close phylogenetic relatives to our isolates. Analysis using the MLST typing scheme for *K. variicola* (available from: https://mlstkv.insp.mx/downloads/, accessed 19 December 2025) revealed that MEZKV13, MEZKV51, and MEZKV52 belong to ST-696 and these three MEZKV isolates are the only three isolates at the time of this report belonging to ST-696 according to the MLST *K. variicola* scheme. Thus, isolates depicting the *K. pneumoniae* ST-2465 multilocus sequence type present in BIGSdb *K. pneumoniae* database were compared to the *K. variicola* isolates from this study.

Since the three STs, ST-5470 (MEZKP33 and MEZKP45), ST-5478 (MEZKP34), and ST-5497 (MEZKP38 and MEZKP48), are described for the first time in this study, no other isolates with the same ST profile are available at the time of writing this report for comparison in BIGSdb-Pasteur; thus, the comparative analysis with isolates from BIGSdb was not performed.

**Figure 9:**
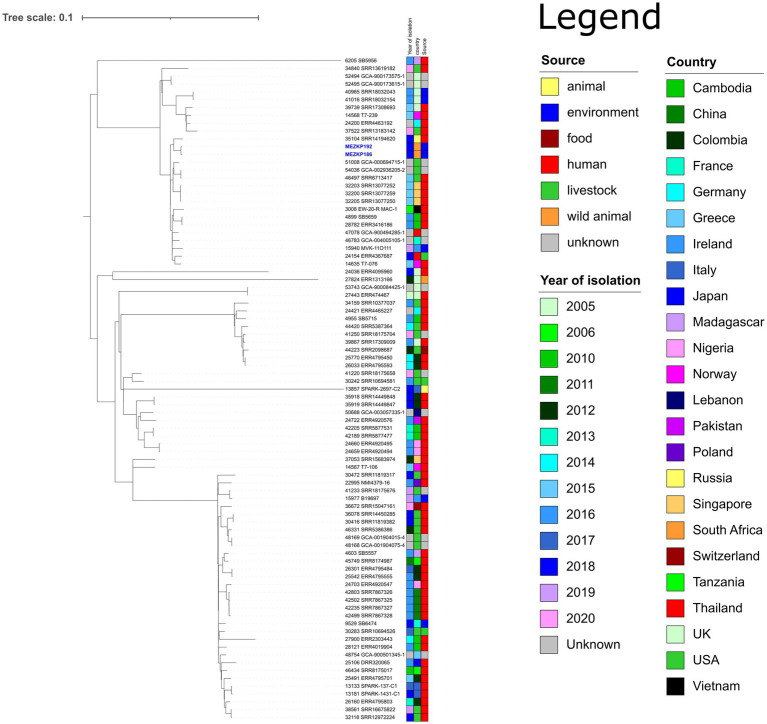
Core genome-based phylogenetic tree of *K. pneumoniae* MEZKP186 and MEZKP192 and all publicly available ST-76 *K. pneumoniae* genomes available in NCBI. The tree was produced in ParSNP and annotated using iTOL v.6.9. Tree was finalized using InkScape 0.9.1 (https://inkscape.org/release/inkscape-0.91/).

The closest phylogenetic relative to our ST-76 (*K. pneumoniae*) isolates (MEZKP186, MEZKP192) was SRR14194620 (BioSample SAMN18679917), isolated from a human blood sample from Russia in 2018. The number of SNPs (234 SNPs to MEZKP186, Supplementary Table 11 and Figure 9) indicates only distant relationship.

**Figure 10:**
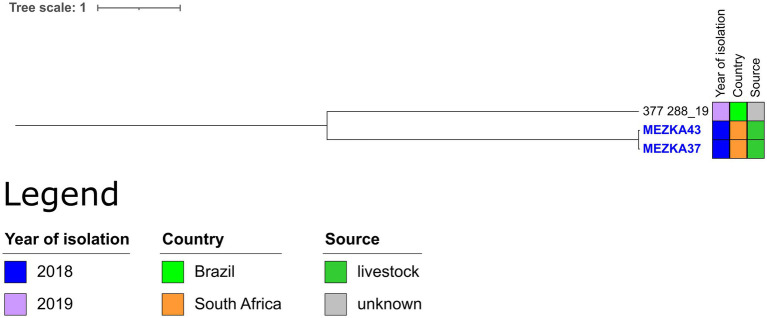
Core genome-based phylogenetic tree of MEZKA43 and MEZKA37 and all publicly available *K. aerogenes* ST-128 genomes available in NCBI. The tree was produced in ParSNP and annotated using iTOL v.6.9. Tree was finalized using InkScape 0.9.1 (https://inkscape.org/release/inkscape-0.91/).

The only *K. aerogenes* ST-128 present in PubMLST (id 377: isolate 288_19) was not phylogenetically related to our ST-128 isolates (MEZKA37, MEZKA43), because it had >10,000 SNPs to our isolates (Supplementary Table 12 and Figure 10).

**Figure 11:**
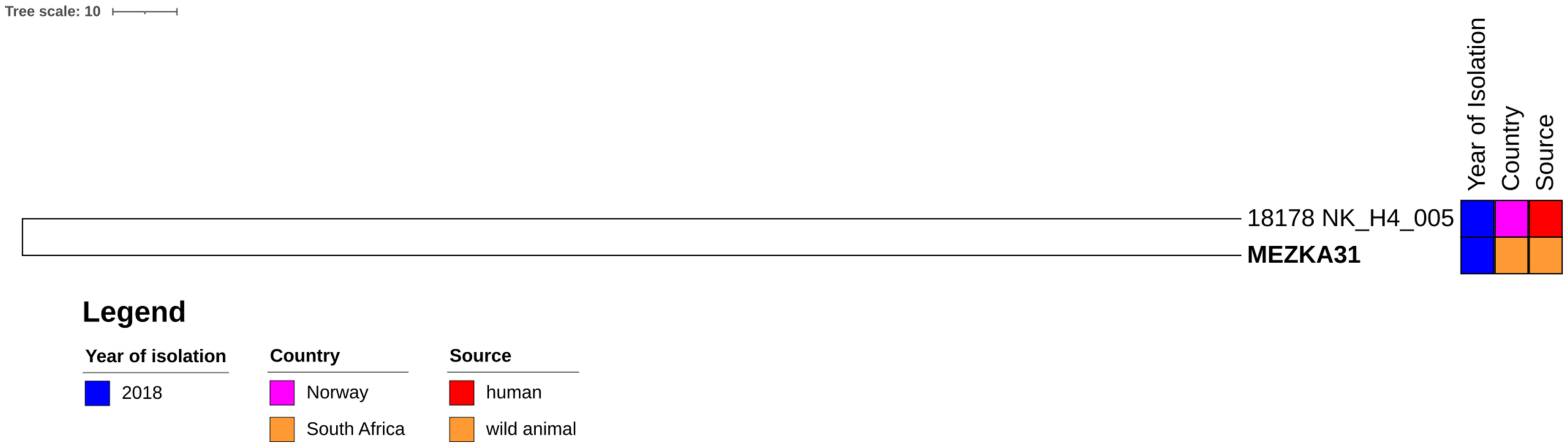
Core genome-based phylogenetic tree of MEZKA31 and the only publicly available ST-494 *K. aerogenes* genome available in NCBI. The tree was produced in ParSNP and annotated using iTOL v.6.9. Tree was finalized using InkScape 0.9.1 (https://inkscape.org/release/inkscape-0.91/).

Only for one ST-494 *K. aerogenes*, sequence data was available (NK_H4_005, human isolate, as of 5th June 2025). This isolate harbored more than 380,000 SNPs compared to our ST-494 *K. aerogenes* isolate (MEZKA31, Supplementary Table 13 and Figure 11), indicating no relationship between these two isolates.

**Figure 12:**
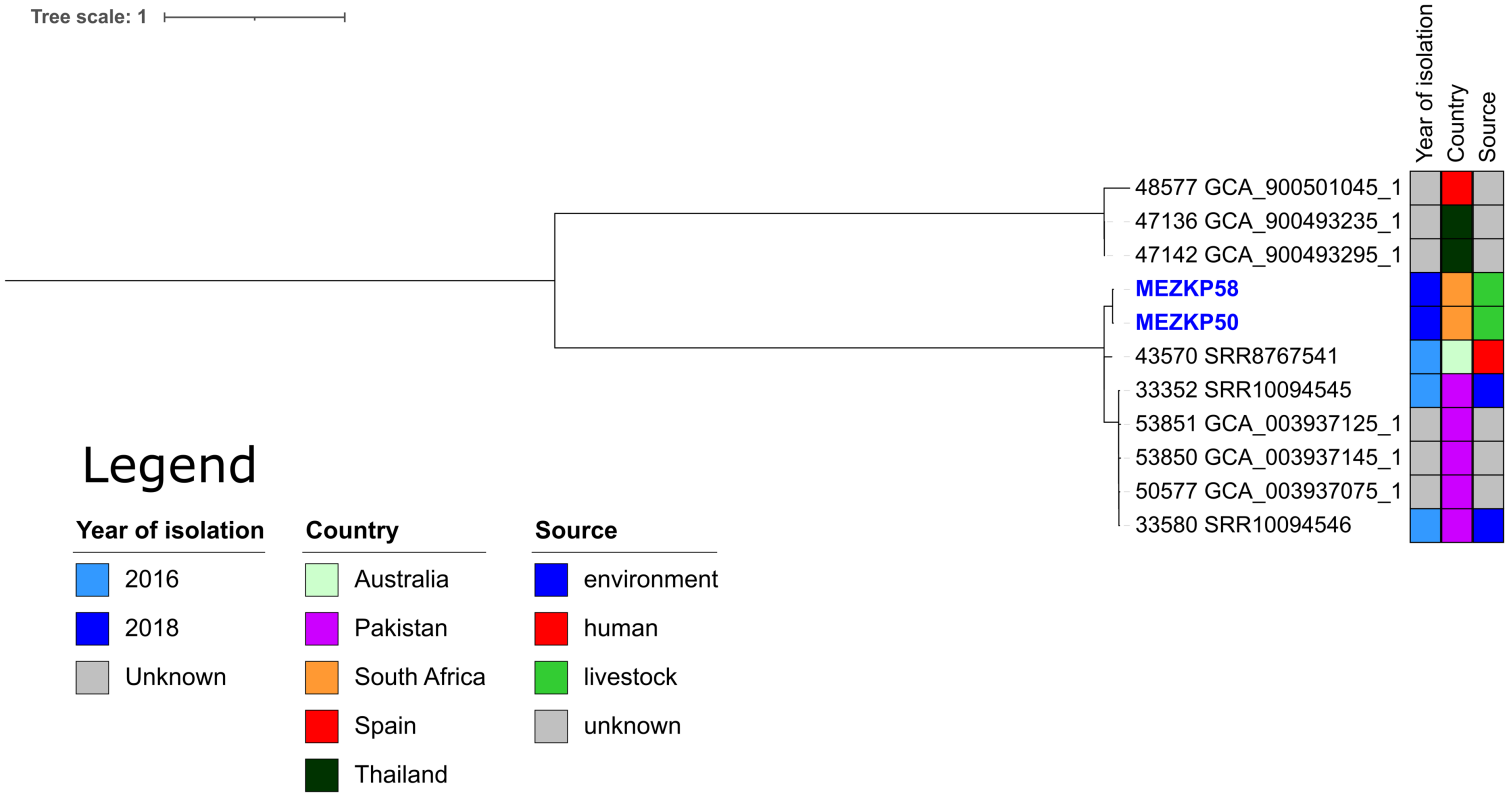
Core genome-based phylogenetic tree of *K. pneumoniae* MEZKP50 and MEZKP58 and all publicly available ST-617 *K. pneumoniae* genomes available in NCBI. The tree was produced in ParSNP and annotated using iTOL v.6.9. Tree was finalized using InkScape 0.9.1 (https://inkscape.org/release/inkscape-0.91/).

The closest phylogenetic relative to our ST-617 *K. pneumoniae* isolates (MEZKP50, MEZKP58) was isolate SRR8767541 (Biosample SAMN11230966), isolated from a human sample, in Australia (363 and 365 SNPs to MEZKP50 and MEZKP58, respectively, Supplementary Table 14 and Figure 12). The number of SNPs (>100) indicates a distant relationship.

**Figure 13:**
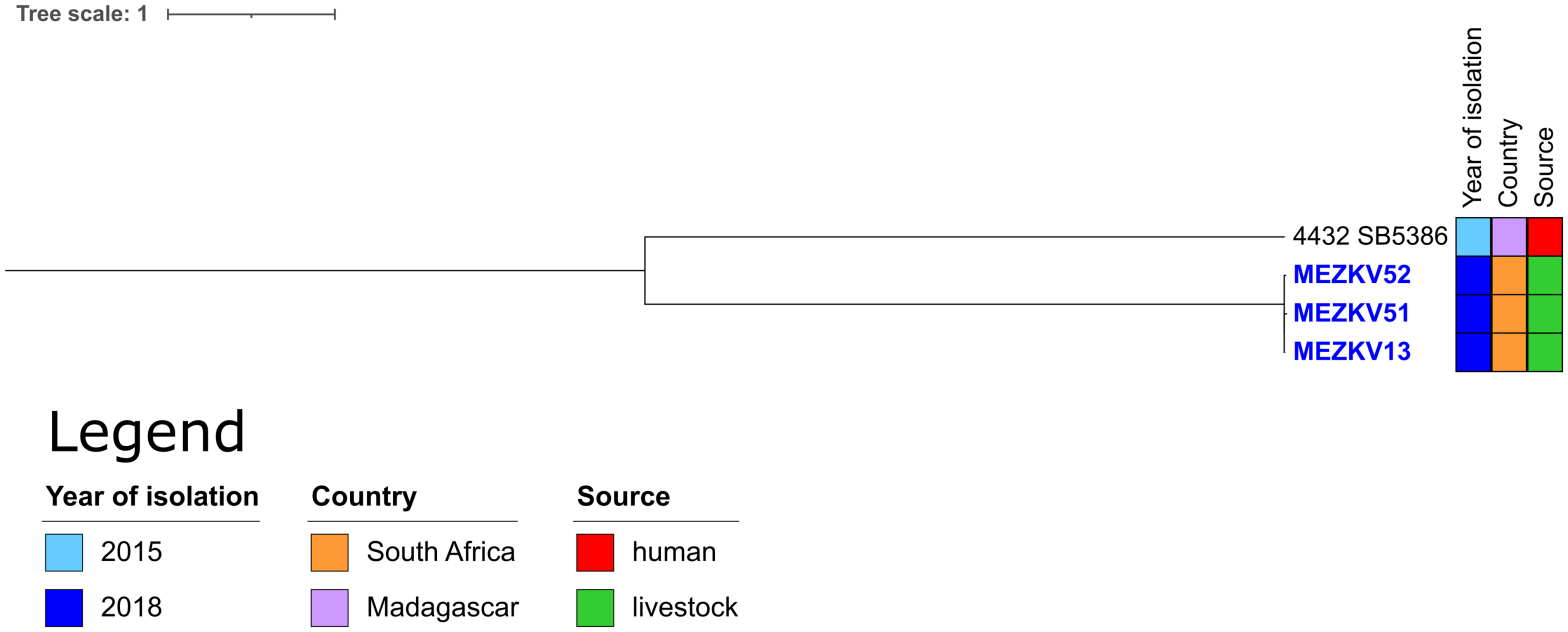
Core genome-based phylogenetic tree of *K. variicola* MEZKV13, MEZKV51, MEZKV52 and the only publicly available ST-2465 *K. variicola* genome available in NCBI. The tree was produced in ParSNP and annotated using iTOL v.6.9. Tree was finalized using InkScape 0.9.1 (https://inkscape.org/release/inkscape-0.91/).

The only ST-2465 (*K. pneumoniae* scheme, but *K. variicola* species) isolate present in the BIGSdb database (SB5386) displayed between 8,413 and 8,481 SNPs to our ST-2465 isolates (MEZKV13, MEZKV51, MEZKV52) indicating no phylogenetic relationship (Supplementary Table 15 and Figure 13). SB5386 was isolated from a human sample in Madagascar.

**Figure 14:**
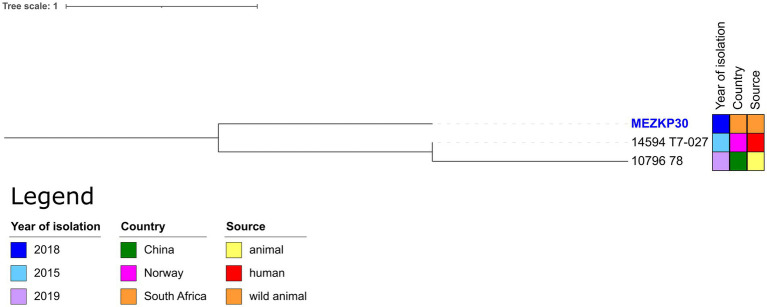
Core genome-based phylogenetic tree of MEZKP30 and all publicly available ST-4039 *K. pneumoniae* genomes available in NCBI. The tree was produced in ParSNP and annotated using iTOL v.6.9. Tree was finalized using InkScape 0.9.1 (https://inkscape.org/release/inkscape-0.91/).

The closest phylogenetic relative to our ST-4039 isolate (MEZKP30) was T7-027, isolated from a human fecal sample in Norway (251 SNPs to MEZKP30, Supplementary Table 16 and Figure 14). Nevertheless, the number of SNPs (>100) indicates only a distant relationship.

### 3.15 Prophage analysis

The 16 *Klebsiella* isolates analyzed contained intact prophages with each genome harboring between one and five prophages. The genomes of MEZKP33 and MEZKP45 isolates contained five prophages. Among the 17 intact prophages, the main prophage detected was PHAGE_Entero_mEp237 (52.9%). The details of the identified prophages including genome size, G+C content, tRNA presence, proportion of hypothetical proteins, and phage identity are summarized in Table 9 and Supplementary Table 17.

**Table 9:** Details of the *in silico* identified prophages integrated in the genomes of the *Klebsiella* isolates including genome size, G+C content, presence of tRNA, proportion of hypothetical proteins, and phage identity.

Isolate ID	Phage	Accession no.	Genome size (kb)	No. of ORFs	Hypothetical protein (%)	G+C (mol%)	tRNA
MEZKA31	PHAGE_Erwini_vB_EhrS_59	NC_048198	50.8	75	24	50.89	0
	PHAGE_Klebsi_3LV2017	NC_047817	22.9	25	0	55.29	0
MEZKA37	PHAGE_Shigel_Sf6	NC_005344	40.3	66	19	47.75	0
MEZKA43	PHAGE_Shigel_Sf6	NC_005344	69.3	60	16	48	0
MEZKP30	PHAGE_Salmon_118970_sal3	NC_031940	48.5	45	8	52.48	3
	PHAGE_Entero_mEp237	NC_019704	54.3	55	9	52.62	0
MEZKP33	PHAGE_Klebsi_ST147_VIM1phi7.1	NC_049451	28.6	30	1	54.52	1
	PHAGE_Salmon_Fels_1	NC_010391	47	60	15	50.33	3
	PHAGE_Erwini_vB_EhrS_59	NC_048198	46.2	66	16	52.06	0
	PHAGE_Entero_mEp237	NC_019704	65.8	53	8	51.66	0
	PHAGE_Klebsi_phiKO2	NC_005857	40	34	5	53.21	0
MEZKP34	PHAGE_Acinet_Bphi_B125	NC_019541	30.5	37	5	51.98	0
	PHAGE_Salmon_118970_sal3	NC_031940	65	78	21	51.31	1
MEZKP38	PHAGE_Salmon_SPN3UB	NC_019545	67.6	80	16	51.98	5
	PHAGE_Salmon_SEN34	NC_028699	54.1	77	17	52.92	1
	PHAGE_Entero_mEp390	NC_019721	46.8	53	7	51.86	1
MEZKP45	PHAGE_Klebsi_ST147_VIM1phi7.1	NC_049451	28.6	30	1	54.52	1
	PHAGE_Salmon_Fels_1	NC_010391	47	57	12	50.32	3
	PHAGE_Erwini_vB_EhrS_59	NC_048198	46.2	67	17	52.06	0
	PHAGE_Entero_mEp237	NC_019704	65.8	53	8	51.66	0
	PHAGE_Klebsi_phiKO2	NC_005857	60	57	17	51.96	0
MEZKP48	PHAGE_Salmon_SPN3UB	NC_019545	61.4	80	24	52.11	5
	PHAGE_Salmon_SEN34	NC_028699	54.1	78	17	52.92	1
	PHAGE_Entero_mEp390	NC_019721	46.8	50	6	51.86	1
MEZKP50	PHAGE_Klebsi_ST437_OXA245phi4.1	NC_049448	40.9	51	3	53.95	1
	PHAGE_Entero_mEp237	NC_019704	54.5	79	20	51.98	1
	PHAGE_Escher_500465_1	NC_049342	37.7	23	3	54.76	1
MEZKP58	PHAGE_Entero_mEp237	NC_019704	55.2	70	14	51.6	1
MEZKP186	PHAGE_Stx2_c_Stx2a_F451	NC_049924	9.6	14	3	54.76	0
	PHAGE_Entero_mEp237	NC_019704	33.6	49	14	54.03	0
	PHAGE_Klebsi_ST512_KPC3phi13.2	NC_049452	29.7	30	1	52.53	0
MEZKP192	PHAGE_Salmon_SEN34	NC_028699	33.6	49	14	54.03	0
	PHAGE_Klebsi_ST512_KPC3phi13.2	NC_049452	23.3	31	1	50.62	0
	PHAGE_Klebsi_3LV2017	NC_047817	32.2	39	6	55.65	0
MEZKV13	PHAGE_Entero_mEp237	NC_019704	52.2	77	30	51.65	0
	PHAGE_Entero_mEp235	NC_019708	33.4	49	7	52.17	0
MEZKV51	PHAGE_Entero_mEp235	NC_019708	33.4	48	7	52.17	0
	PHAGE_Entero_mEp237	NC_019704	52.2	77	30	51.65	0
MEZKV52	PHAGE_Entero_mEp235	NC_019708	35.1	44	7	52.92	0
	PHAGE_Entero_mEp237	NC_019704	47.9	69	22	51.33	0

## 4 Discussion

Animals (wild, companion, livestock, seafood, and fish), foods (fresh produce, fruits, cooked foods, and retail foods), and the environment (wastewaters, hospital environment, soil, rivers, and oceans) ecosystems constitute significant reservoirs of resistant bacteria involved in human and animal infections (Heljanko et al., 2024; Dossouvi and Ametepe, 2024; Richter et al., 2024; Kot and Witeska, 2024). Although many previous genomic surveillance studies have focused on clinical or human-derived *Klebsiella* isolates (Salvador-Oke et al., 2024), our study contributes rare comparative genomic data derived from livestock and environmental samples in South Africa. This livestock-associated genomic insight reveals distinct phylogenetic clustering, antimicrobial resistance, and virulence gene patterns that are less characterized in public datasets, especially for *K. aerogenes* and *K. variicola.*

Molecular epidemiological characterization of multidrug-resistance in *K. pneumoniae* obtained from human infections have been well studied. However, limited data are available about the ecology and population of *Klebsiella* species including *K. pneumoniae* in various wild animals, livestock and other non-human hosts (Wyres et al., 2020a).

The application of WGS-based characterization of *Klebsiella* spp. isolated from animals in South Africa is scarce. The present study is the first to report the detection and WGS-based analyses of *Klebsiella* spp. isolated from different animals including wild birds, livestock, and the environment.

Results of the current study demonstrated that farm animals might be an important source of multidrug resistant *Klebsiella* species including the human pathogen *K. pneumoniae*. In the present study, 16 *Klebsiella* spp. isolates were identified out of 197 samples collected from farm animals and surroundings accounting for a prevalence rate of 8.1%. Among the species of the isolates confirmed using WGS-based analysis were *K. pneumoniae* (5%, *n* = 10), *K. aerogenes* (1.5%, *n* = 3, two isolates from pig samples and one isolate from wild duck), and *K. variicola* (1.5%, *n* = 3, chicken). Three *K. pneumoniae* isolates were recovered from water samples in the farm and the surroundings, two isolates were from pigs and two from sheep, and one isolate from each of a cow, chicken and wild bird. The current study is the first to report the phenotypic and genomic surveillance for *Klebsiella* spp. recovered from samples collected from livestock, wild birds, their surrounding and environment in South Africa. A limited number of studies from Africa are available in literature on the detection of *Klebsiella* species in food animals in a *One Health* context, including a study from Ghana on the detection of antimicrobial-resistant *Klebsiella* isolates from human, farm animals, and environmental sources (Calland et al., 2023). Albeit the numbers of *Klebsiella* isolates detected in the Ghanaian study were higher than in our study (64.7% vs. 8.1%), the species distribution among sequenced isolates were in part similar to the results from our study, as *K. pneumoniae* was the most common *Klebsiella* species in Ghana (65% compared to 62.5% in this study, 10 out of 16). Not comparable were the results for *Klebsiella quasipneumonia* subspecies *similipneumoniae* (24% in the Ghanaian study) (Calland et al., 2023), which was not detected in our study at all. The low percentage rates of the detection of *Klebsiella* spp. in the current study as compared to the study from Ghana may be attributed to differences in the number of samples tested, geographic location, and host.

A study conducted in South Africa on food animal production-associated isolates from an abattoir environment and wastewater reported that *E. coli* and *K. pneumoniae* were the most abundant species (Heljanko et al., 2024). In the present study, *K. pneumoniae* accounted for 10 (62.5%) out of 16 *Klebsiella* isolates, which is consistent with these findings.

Our results were comparable with those from another study performed in Germany that reported the detection of 94 *Klebsiella* spp. isolates from vegetables, food sources, pets, livestock, and wild animals of which most isolates (*n* = 58; 61.7%) were from pigs, pork, cattle, and milk (Klaper et al., 2021). The species distribution in the German study (71.3%, *K. pneumoniae*, 4.3% *K. variicola* subsp. *variicola* and 1.1% as *K. quasipneumoniae* subsp. *Similipneumoniae*) (Klaper et al., 2021) correlates with our findings except for *K. quasipneumoniae*, which was not detected in our study.

The detection of multidrug resistance and virulence genes in *Klebsiella* spp. isolates from different sources and regions has been reported.

Wild animals are a potential source of *Klebsiella* infection which can spill over to other hosts including humans. In the present study, two *Klebsiella* spp. isolates (one *K. pneumoniae* MEZKP30 and one *K. aerogenes* MEZKA31) were isolated from oral samples obtained from wild ducks in Durban, KwaZulu-Natal Province. While MEZKP30 was resistant to three classes of antibiotics, MEZKA31 was phenotypically resistant to four classes of antibiotics (Table 4) and both isolates showed MDR pattern. Our findings correlate to previous studies, as recently, the emergence of MDR *K. pneumoniae* in wild animals in Africa was reported. Two MDR *K. pneumoniae* isolates were detected in 53 fecal samples obtained from giraffes, elephants, and rhinos imported from Africa before they entered China (Yang et al., 2024).

In the present study, the 16 *Klebsiella* isolates demonstrated MDR pattern. This coincides with a previous study in South Africa, where all *K. pneumoniae* isolates detected were multidrug resistant (Heljanko et al., 2024).

The genotypic and phenotypic discordance was previously reported among different bacteria (Saxenborn et al., 2021; Yee et al., 2021; Kawser et al., 2025; Eladawy et al., 2024; Shrivas et al., 2025). The genotype and phenotype mismatch is a well-recognized phenomenon and discrepancies between genetic determinants (genotypes) detected in various bacteria, e.g., *Salmonella, E. coli, Campylobacter;* and *K. pneumoniae* using whole-genome sequencing and their phenotypes are well-documented and have been reported elsewhere in literature (Ball et al., 2020; McDermott et al., 2016; Urmi et al., 2020; Adhikari et al., 2025; Zhao et al., 2016; Tumeo et al., 2025; Thorpe et al., 2022).

No colistin or carbapenem resistance encoding genes were identified in the genomic sequences of the 16 *Klebsiella* isolates reported in the current study. This observation is consistent with findings from other studies in South Africa and Nepal (Heljanko et al., 2024, Hosuru Subramanya et al., 2020, Subramanya et al., 2021). However, in the present study, the majority of the *Klebsiella* isolates (93.75%, *n* = 15) were phenotypically resistant to colistin with MIC as high as 32 μg/mL. Differences between the phenotypic and genotypic resistance patterns of the current isolates can be explained due to other underlying colistin resistance mechanisms including low permeability.

This discrepancy may be explained by the presence of resistance mechanism(s) that have not yet been described or due to sequence gaps from the draft genomes that prevented the detection of one of the known colistin resistance genes. This indicates a limitation of the WGS-based antimicrobial resistance detection approach and necessitates further analysis of the possible mechanism(s) of colistin and carbapenem resistance in the isolates. In order to fully investigate the underlying phenotypic colistin and carbapenem resistance mechanisms of the isolates will require further investigations which are beyond the scope and objectives of the current study.

All the 16 *Klebsiella* isolates were resistant to imipenem with MIC ≥8 μg/mL. One *K. aerogenes*, one *K. variicola* and two *K. pneumoniae* isolates were susceptible to meropenem. Out of the 16 *Klebsiella* isolates, five *K. pneumoniae*, one *K. aerogenes*, two *K. variicola* isolates were resistant to both meropenem and imipenem. One *K. aerogenes* and three *K. pneumoniae* isolates showed intermediate susceptibility to meropenem as shown in Table 4. The reduced susceptibility of the *Klebsiella* isolates to carbapenems may be explained by the detection of chromosomal mutations in *ompK*36, *ompK*37, and *acrR* which are predicted to play a role in the phenotypic resistance against carbapenems as previously reported (Binsker et al., 2024; Legese et al., 2022; Schneiders et al., 2003; Ruan et al., 2024). The 16 *Klebsiella* isolates harbored various combinations of these mutations, and the specific combinations and positions of these mutations varied across the genomes, as shown in Supplementary Table 6. The outer membrane porin proteins are key entry route for β-lactam antibiotics including carbapenems, and the loss or modification of OmpK36 reduces antibiotic influx, which contributes significantly to antibiotic resistance, particularly to carbapenems as previously reported (Wong et al., 2022).

Our results of colistin resistance phenotype, despite an apparent absence of colistin resistance genes are in contrast to a study in the North-West Province of South Africa (Ramatla et al., 2024), where PCR-based methods were used to determine the presence of ESBL and colistin resistance genes in *K. pneumoniae* isolated from broiler chickens. In that study, 47.1% of the 17 detected *K. pneumoniae* isolates carried *mcr-1* gene but not *mcr-2* (Ramatla et al., 2024), albeit all PCR products were not subjected to sequencing analysis to further identify the ESBL types and to confirm the PCR results of the reported *mcr* genes.

It was recently reported that Kleborate did not detect colistin resistance mechanism in three *K. pneumoniae* isolated from pigs and *K. aerogenes* isolated from a goat (Thorpe et al., 2022). This was not unexpected since many *mcr* variants are not included in the Kleborate database and colistin resistance can also be conferred through mutations responsible for membrane synthesis as previously reported (Ayoub, 2020). It was previously reported that a relatively high discordance of antibiotic susceptibility results was observed between phenotypic antimicrobial susceptibility testing and WGS-based-antimicrobial susceptibility testing, regardless of the bioinformatics analysis tool used (Saxenborn et al., 2021). One of the main limitations of genotypic methods is that the detected genetic determinants are not necessarily expressed and translated into phenotypic resistance (Saxenborn et al., 2021).

The genotype–phenotype discordance may be explained by the presence of natural resistance not found genotypically or the presence of a currently undescribed allelic variant related to resistance as previously reported (Levin and Rozen, 2006; Saxenborn et al., 2021; Jia et al., 2025).

In our study, no ESBL genes have been detected, which is in contrast to another study from South Africa which reported the detection of ESBL determinants *bla*_CTX-M_ and *bla*_OXA_ in *K. pneumoniae* isolated from bovine fecal and raw beef samples in the North-West Province in South Africa (Montso et al., 2019).

In the present study, three *K. pneumoniae* isolates MEZKP34, MEZKP186, and MEZKP192 isolated from water and wastewater samples in the farm surroundings were both phenotypically and genotypically designated as MDR strains. This is consistent to results of other reports from South Africa (Fadare et al., 2023). Moreover, multidrug-resistant and clinically relevant *K. pneumoniae* isolated from surface water samples have been detected in Germany, also consistent with our data (Falgenhauer et al., 2019) emphasizing that environmental and wastewater monitoring plays a crucial role in stemming the global spread of AMR (Honda et al., 2023; Pruden et al., 2021). *K. pneumoniae* is one of the ESKAPE pathogens (*Enterococcus faecium, Staphylococcus aureus, Klebsiella pneumoniae, Acinetobacter baumannii, Pseudomonas aeruginosa*, and *Enterobacter* species), which is known for its excessive intraspecies and interspecies exchange of genetic information with the environment and other bacteria (Wyres and Holt, 2018).

In the current study, the detection of phenotypically carbapenem-resistant *K. pneumoniae* were isolated from environmental wastewater samples which did not harbor apparent carbapenem resistance genes. This result is in contrast to a study on *Klebsiella* spp. isolated from different environmental settings including wastewater hospital effluents, surface and irrigation waters, vegetables, soil, and farms in the Eastern Cape Province, South Africa (Ebomah and Okoh, 2020), where 43% of the 234 confirmed *Klebsiella* spp. isolates carried *bla_KPC_*, *bla_OXA-48-like_*, and *bla_NDM-1_* carbapenem-resistance genes (Ebomah and Okoh, 2020). Tigecycline is one of the last-resort antibiotics which are used to treat severe and life-threatening infections caused by MDR Gram-negative bacteria (Rodríguez-Baño et al., 2018; He et al., 2019). Notably, all the 16 *Klebsiella* isolates tested in the current study were susceptible to tigecycline. None of the tested isolates harbored any *tet*(X) variants or mutations associated with tigecycline resistance. These findings contrast other studies which reported the dissemination and increased detection of tigecycline-resistant *Klebsiella* isolated from humans, food, companion animals, livestock, and environmental samples (Liu et al., 2022; Zhang et al., 2022; Wu et al., 2026; Kürekci et al., 2026).

In the present study, *Klebsiella* species were not detected in the human swab samples obtained from the hands, axilla and groin of the farm workers. These results are in contrasts to findings from a study performed previously in South Africa, where *K. pneumoniae* isolates were detected in samples obtained from the hands of veterinary students at a veterinary academic hospital (Sebola et al., 2024) and a study from Egypt, where 13.3% *Klebsiella* spp. and 6.6% *K. pneumoniae* isolates were recovered from 30 human hand swab samples (Khalefa et al., 2025).

In the present study, the two equine fecal samples and the feed samples were negative for *Klebsiella* spp. This could be attributed to the small number of samples tested and is in contrast to earlier studies. For example, in another study, 15% of horse and 8% of feed samples were positive for *Klebsiella* spp., of which 8.3% were determined to be *K. pneumoniae* in horses and 2% *K. pneumoniae* in feed samples as previously reported (Khalefa et al., 2025). In another study from Egypt, *K. pneumoniae* was detected in 4.4% of horse samples (Arafa et al., 2022).

In the present study, three *K. pneumoniae* isolates, MEZKP33 and MEZKP38 assigned to novel sequence types ST-5470 and ST-5497, respectively, and MEZKP192 belonging to ST-76 were designated as MDR. To the authors’ knowledge, this is the first report, at the time of writing this manuscript, among South African *K. pneumoniae* isolates recovered from non-human sources (wastewater and livestock) in KwaZulu-Natal and Eastern Cape Provinces on the detection of multidrug-resistant *K. pneumoniae*.

In the present study, none of the genes associated with hypervirulence (*rmpA*, *rmpA2*, *iucA*, *irp2*, K1 or K2 capsule type) were detected in the 16 *Klebsiella* genomes when analyzed *in silico* using Kleborate and BLASTN of primers targeting gene sequences characteristic for hypervirulence (Rodríguez-Medina et al., 2024).

In a previous study, 5 out of 10 *K. pneumoniae* isolates demonstrated hypermucoviscosity (Soto et al., 2020). It was reported that the presence of HMV phenotype was linked with the appearance of invasive syndromes and has been correlated with the abscess formation in community-acquired *K. pneumoniae* bacteremia (Yu et al., 2006). In the present study, MEZKP33, MEZKP38, and MEZKP192 demonstrated HMV based on the *in vitro* string test results, and these three isolates were resistant to ampicillin. Similar results were obtained by Lee et al., where all of the 140 HMV isolates showing invasive syndromes were resistant to ampicillin (Lee et al., 2010). In the present study, MEZKP192, a hypermucoviscous strain, was isolated from wastewater sample collected from proximity to local hospital and livestock farm, similar to a previous study from Germany (Falgenhauer et al., 2019). The hypermucoviscosity of *Klebsiella* has been linked to the formation of a mucoid capsule, one of the virulence factors attributable to the evasion of host innate immunity and AMR (El Fertas-Aissani et al., 2013).

Experiments demonstrating hypervirulence of the reported *Klebsiella* strains in the present study were not performed. One of the limitations of the current study is regarding the complete characterization, including *in vivo* model assays that would definitively determine whether the strains are hypervirulent. Further *in vivo* investigations including pathogenicity tests in mice are required to explore the role of the hypermucoviscosity of the current isolates in the virulence and their underlying molecular mechanisms.

It was reported that MDR-hypervirulent *Klebsiella* spp. are known to be undergoing further evolution to produce phenotypically novel strains (Gu et al., 2018; Wyres et al., 2020a, 2020b; Dong et al., 2018; Russo and Marr, 2019; Dong et al., 2022). This may explain that MEZKP33 and MEZKP38 belong to novel sequence types ST-5470 and ST-5497, respectively. Importantly, the identification of five isolates belonging to three novel sequence types (ST-5470, ST-5478, and ST-5497) (Table 7 and Supplementary Table 9) for *K. pneumoniae* in the present study underscores the ongoing evolution of *Klebsiella* populations in non-human reservoirs. These findings highlight the need for routine whole-genome surveillance in animals and the food chain to monitor the emergence of genetically distinct MDR clones that may eventually spill over into human populations.

The *Klebsiella* isolates in the current study were recovered from different sources including wild birds, water, soil, and livestock but also in the neighborhood and proximity to farm owners and slaughterhouses, indicating a possible transmission potential of the bacteria into food chain, other animal populations, and humans, which requires further large-scale investigation.

The rural population of South Africa attained a value of 18,956,858 people in 2024.^10^ In South Africa, approximately 30% of the 63 million people of South Africa live in rural areas and are engaged in farming practices. The majority of farming practices is subsistent in nature, and the crop is mostly integrated with livestock (cattle, goat, pig, and poultry), wildlife animals and birds. In South Africa, livestock is exposed to antibiotics via feed additives or treatments. Irrational use and imprudent application of antibiotics in agricultural and livestock production sectors without prior consultation of a veterinarian in South Africa significantly contribute to the development and emergence of resistant pathogens including MDR *Klebsiella* spp.

In the present study, such a high percentage of MDR *Klebsiella* spp. in livestock production settings underlines the potential risks to human and animal health due to possible dissemination of virulent and resistant *Klebsiella* isolates or their resistance genes via farm-to-fork transmission or environmental routes. In the present study, the 16 *Klebsiella* strains were isolated from different sources including food animals and the environment raising concerns that *Klebsiella* strains could be transmitted to humans through farm-to-fork route. *Klebsiella* is an important foodborne pathogen and *K. pneumoniae* has been isolated from various foods including raw vegetables, powdered infant formula, meat, fish, and retail foods as previously reported (Sun et al., 2010; Puspanadan et al., 2012; Zhang et al., 2018; Davis and Price, 2016; Kim et al., 2015; Overdevest et al., 2014; Haryani et al., 2007). *K. pneumoniae* was previously reported to cause increasing numbers of foodborne outbreaks worldwide (Bi and Xu, 2013; Calbo et al., 2011; Yu and Zhou, 2013).

None of the *Klebsiella* isolates in the present study harbored a carbapenem resistance gene when the genome sequences were analyzed using either ResFinder-2.2 or Kleborate (Supplementary Table 5), albeit the isolates showed phenotypic resistance to imipenem (100%) and meropenem (50%). This finding is different from results of other studies which previously reported that carbapenemase- and ESBL-producing *Klebsiella pneumoniae* were detected in humans and livestock in rural areas in Cambodia (Atterby et al., 2019), from chickens in Bangladesh (Hasan and Swedberg, 2022), from broiler liver samples in Algeria (Chenouf et al., 2021), and from bovine bulk milk samples in Italy (Bonardi et al., 2023).

In the present study, genotypic analysis of AMR genes in the 16 *Klebsiella* genomes revealed the presence of β-lactam resistance genes [*bla*_SHV_ variants (*bla*_SHV-11-like_*, bla*_SHV-40/56/79/85/89-like_*, bla*_SHV-110/81-like_*, bla_SHV-119_, bla*_SHV-164/59-like_), and *bla*_LEN-16-like_], fosfomycin resistance genes (*fosA*, *fosA5*, *fosA6*, or *fosA7*) and quinolone resistance genes (*oqxA* and *oqxB*). The OqxAB multidrug efflux pump genes (*OqxA* and *OqxB* or their homologues) were detected in the ten *K. pneumoniae* and the three *K. varicola* isolates in the current study. This finding is concordant with the MDR phenotypic results. Furthermore, the OqxAB multidrug efflux pump has been increasingly reported in *Enterobacteriaceae* and it confers resistance to a wide range of antimicrobial agents (Wong et al., 2015; Maurya et al., 2019; Xu et al., 2021; Bharatham et al., 2021). None of the *bla_SHV_* gene variants are classified as ESBLs. This observation contrasts with previous reports which frequently identified ESBL- or carbapenemase-producing *Klebsiella* strains as previously reported elsewhere (Sharew et al., 2025; EFSA BIOHAZ Panel (EFSA Panel on Biological Hazards) et al., 2025; Ouchar Mahamat et al., 2021; Hosuru Subramanya et al., 2020; Atterby et al., 2019; Subramanya et al., 2021; Bonardi et al., 2023; Kayama et al., 2023; Chenouf et al., 2021). Thus, the results of our study only in part correlate to studies performed on *Klebsiella* spp., where MDR, but in contrast ESBL-producing *Klebsiella* isolates harboring *bla*_CTX-M_, *bla*_OXA_, *bla*_TEM_, *bla*_SHV_, and *bla*_CMY_ were previously reported in animals, food, and the environment (Wareth and Neubauer, 2021). These findings emphasize that the phenotypic and genotypic patterns of resistance differ among antibiotic groups, hosts, and locations (Kayama et al., 2023; Klaper et al., 2021; Thorpe et al., 2022; Kot and Witeska, 2024).

It was previously reported that chromosomal mutations in *ompK*36, *ompK*37, and *acrR* are predicted to play a role in phenotypic carbapenem, cephalosporin, and fluoroquinolone resistance, respectively (Binsker et al., 2024; Legese et al., 2022; Schneiders et al., 2003; Ruan et al., 2024).

*AcrR* is a repressor of AcrA/AcrB/TolC multi-drug efflux pump and *AcrR* mutations result in high level antibiotic resistance as previously reported (Suzuki et al., 2014). However, the specific combinations and positions of these mutations varied across the 16 *Klebsiella* isolates, as detailed in Supplementary Table 6 (Wong et al., 2022).

Biofilm formation is a well-recognized and prevalent trait among *Klebsiella* isolates, especially those associated with persistence, antimicrobial resistance, or host colonization. The ability to form biofilms is a crucial virulence trait for several microorganisms, including *Klebsiella pneumoniae* as detailed elsewhere (Guerra et al., 2022). In the present study, the 16 (100%) *Klebsiella* isolates demonstrated the formation of a biofilm *in vitro*. Importantly, while all the tested *Klebsiella* strains in the current study demonstrated measurable biofilm formation under the assay conditions used, the magnitude of the biofilm production varied significantly among isolates. The biofilm formation was found to be highest in isolate MEZKA31 (*K. aerogenes*) followed by MEZKV13 (*K. variicola*). Both HMV and non-HMV isolates were found to produce biofilms. Interestingly, the non-HMV isolates had higher biofilm forming capacity when compared to the HMV isolates.

Our results coincide with a study on *K. pneumoniae* isolated from sea lions and seals in the United States. The study demonstrated that though both HMV and non-HMV isolates were able to produce biofilms. The biofilms produced by the non-HMV isolates were significantly higher than those produced by the HMV isolates (Soto et al., 2020). All the antibiotic-resistant isolates demonstrated the ability to form biofilms, particularly those resistant to ampicillin. This ampicillin resistance may be attributed to the enhanced biofilm forming ability of the *Klebsiella* isolates as previously reported (Kumon et al., 1994, Pinto et al., 2020, Wang et al., 2020; Guerra et al., 2022; Kabir et al., 2025, Lamichhane et al., 2024), although this association requires further investigation. Similar results were reported by Anderl et al. (2000), where ampicillin showed poor penetration into biofilms produced by ampicillin resistant wild-type *K. pneumoniae in vitro*. A similar finding was reported by Nirwati et al. (2019), where all 962 biofilm producing *K. pneumoniae* isolates recovered from clinical samples demonstrated 100% resistance to ampicillin.

Multiple virulence genes were reported in *K. pneumoniae* genome which affect bacterial adhesion, colonization, invasion, and adherence to host organs or tissue surfaces (Han et al., 2025). Furthermore, several studies have reported numerous biofilm formation associated genes such as those involved in the production of aerobactin (*iutA*), allantoin (*allS*), type I fimbriae (*fimA* and *fimH*), type III fimbriae (*mrkA* and *mrkD*), capsular polysaccharides (*cpsD, treC, wabG, wcaG, wzc, k2A*, and *wzyK2*), quorum sensing (*luxS*), and colonic acid (*wcaJ*) (Ashwath et al., 2022; Zheng et al., 2018; Chen et al., 2020).

Virulence gene analysis showed variable patterns among the *Klebsiella* isolates, with possible species-related trends. All isolates carried the efflux determinant *acrAB*, supporting intrinsic resistance and environmental persistence (Jang, 2023). Adherence- and iron acquisition-related genes (*ompA*, *yagV–yagZ*, *ykgK*, *entA*, *entB*, *fepC*) were prevalent in *K. pneumoniae* and *K. variicola*, suggesting conserved mechanisms for attachment and nutrient uptake (Folgori et al., 2021; Lan et al., 2025). Some *K. variicola* carried *rcsAB*, linked to capsule regulation (Huang et al., 2006). *K. aerogenes* tended to encode motility (*flg*, *fli*) and siderophore (*iroB*, *iroN*) genes, indicating ecological flexibility (Chilcott et al., 2000; Lan et al., 2025). Collectively, these features reflect shared adherence and iron acquisition strategies with species-level genomic variation shaped by environmental adaptation.

In the present study, six *K. pneumoniae* isolates contained IncF, IncR, and Col plasmid replicon types, with IncFII_1_pKP91 predominance. Two isolates MEZKP192 and MEZKP186 recovered from wastewater samples carried six and seven different plasmid replicons, respectively.

The emergence of multidrug and carbapenem-resistant *K. aerogenes* and *K. variicola* isolates increased research interests to explore these opportunistic pathogens from non-human sources such as food animals and the environment. In the present study, *K. aerogenes* and *K. variicola* isolates did not carry any plasmid replicons. These results contrast to other studies, where the most common type of plasmid replicon identified in *K. aerogenes* was the ColRNAI as previously reported (Passarelli-Araujo et al., 2019; Morgado et al., 2024) and other studies that reported the detection of plasmid replicons in *K. variicola* isolates from different sources (Duran-Bedolla et al., 2023; Ota et al., 2022; Rodríguez-Medina et al., 2020). In contrast to our study, *K. aerogenes* clinical isolates were reported in recent studies. *K. aerogenes* is an emerging pathogen which has been associated with multidrug and carbapenem-resistant infections in humans as previously reported (Morgado et al., 2024; Merhi et al., 2023).

The prophages identified in the genomes of the 16 *Klebsiella* strains in this study exhibited substantial genomic variation, with genome sizes ranging from 9.6 to 69.3 kb, consistent with previous large-scale analyses of *K. pneumoniae* (Marques et al., 2021). This heterogeneity reflects the mosaic architecture of mobile genetic elements that shape the *Klebsiella* genome (Marques et al., 2021; De Koster et al., 2022). A notable proportion of prophage-encoded ORFs were annotated as hypothetical proteins, reaching up to 30% in some phages. This highlights the novelty of these regions. Similarly, tRNA genes were present in nearly all unique prophages, with some encoding up to five. While their exact function remains uncertain, tRNAs have been proposed to enhance translational efficiency during lysogeny or lytic induction (Marques et al., 2021; Mohammadi et al., 2023). The genome sequences of temperate bacteriophages (prophage regions) are well known as hotspots for genetic recombination and gene acquisition, often carrying mobile elements and antimicrobial resistance genes (De Koster et al., 2022; Kang et al., 2023; Marques et al., 2021; Wang et al., 2019; Baliga et al., 2021; Bleriot et al., 2020). These features contribute to genomic plasticity and may offer selective advantages such as antibiotic resistance (Shen et al., 2020; Wang et al., 2019). Although resistance genes were not directly assessed in this study, the consistent detection of intact prophages across all isolates supports their ubiquity and potential roles in host adaptation and diversification (Marques et al., 2021; Bleriot et al., 2020).

Antimicrobial resistant pathogens are spreading across all ecosystems. Antibiotic resistance is a constantly evolving problem with chronic and endemic patterns (MacIntyre and Bui, 2017), causing global pressing economic and health crisis with significant potential to impact animal, public and environmental health, and food and water biosecurity (Djordjevic et al., 2024). Antimicrobial resistance endangers progress in medicine, food production, and life expectancy (Javvadi and Mohan, 2024). Antimicrobial resistance is a threat to global health and security, driven by the intensive use and application of antibiotics and antimicrobial agents in medicine, livestock and agriculture. *K. pneumoniae* is an important pathogen isolated from hospitals, animals and the environment. The global MDR problems of *K. pneumoniae* and other *Klebsiella* species of clinical significance are of grave concerns, and the antimicrobial treatment options for such infections are becoming limited (Rice, 2010).

Whole-genome sequencing and metagenomic approaches have improved deciphering the complexity of AMR and such approaches are providing useful data to help develop mitigation measures to delineate, control AMR in animals and humans (Struelens et al., 2024; Wheeler et al., 2023). Thus, these findings emphasize the importance of genomic surveillance to monitor the prevalence and spread of antimicrobial and virulence genes from livestock and environment to humans.

## 5 Conclusion

The findings of the current study highlight the significance of molecular and genomic surveillance studies in monitoring and tracking antimicrobial resistance among *Klebsiella* spp. isolated from food animals and the surrounding environment. The One Health principles are integral to AMR research programs to mitigate the risks associated by non-clinical (livestock, environmental, and wildlife) reservoirs of AMR through close monitoring and genomic surveillance efforts. The detection of MDR *Klebsiella* spp. in the present study poses risks on multiple levels including the potential of sporadic transmission from livestock to farm workers. In addition, this study was an in-depth genome-wide analyses of *Klebsiella* species using WGS analyses, as well as providing valuable data and new insights into the complex phenotypic and genotypic antimicrobial resistance and pathogenesis of livestock-associated *Klebsiella* species. The identification of isolates belonging to three novel sequence types (ST-5470, ST-5478, and ST-5497) for *K. pneumoniae* in the present study underscores the ongoing evolution of *Klebsiella* populations in non-human reservoirs. These findings revealed that food-producing animals, wild animals, wastewater and environmental samples hold potential for dissemination of virulent and MDR *Klebsiella* species emphasizing the critical need for antimicrobial resistance surveillance studies. Moreover, the detection of pathogenic and resistant *Klebsiella* spp. including MDR strains, underscore the importance of implementing strict jurisdictions on prudent use of antibiotics in animal productions in South Africa. Altogether, the results highlight the importance of implementing the *One Health* principles in containing antimicrobial resistance dissemination in animal-origin resistant pathogens.

## Data Availability

The whole genome sequencing project has been deposited at GenBank/NCBI under BioProject PRJNA716986, BioSample accession numbers SAMN18762340- SAMN18762355, and GenBank accession numbers JAGWEA000000000- JAGWEM000000000 and JAGWDX000000000- JAGWDZ000000000. The sequences have been submitted to the Sequence Read Archive (SRA) under the accession numbers SRR14584973- SRR14584988. Ten *Klebsiella pneumoniae* and three *Klebsiella variicola* were submitted to *Klebsiella* Pasteur MLST database under ID numbers 15853-15865. Three *Klebsiella aerogenes* were submitted to *Klebsiella aerogenes* PubMLST database under ID numbers 986-988. The data generated or analyzed during this study are included in this published article and its associated Supplementary files.

## Footnotes

1
https://pubmlst.org/organisms/klebsiella-aerogenes

2
https://bigsdb.pasteur.fr/klebsiella/

3
https://mlstkv.insp.mx/

4
https://github.com/tseemann/abricate

5
https://www.atcc.org/products/13048

6
https://www.atcc.org/products/13883

7
https://www.bv-brc.org/

8
http://stothard.afns.ualberta.ca/cgview_server/

10 Available at: https://www.globaldata.com/data-insights/macroeconomic/the-rural-population-of-south-africa-478677/. Accessed 13 May 2025

## Supplementary material

SUPPLEMENTARY FIGURE 1 The string test used to determine hypermucoviscosity. The formation of string from stretching the colonies of *Klebsiella* isolates on sheep blood agar plates (pictured in photos) indicates a hypermucoviscous isolate. The degree of hypermucoviscosity is based on the length (in mm) of the string.

SUPPLEMENTARY FIGURE 2 Distance-based phylogeny. A distance-based phylogeny method (Criscuolo, 2019) was used to confirm the taxonomic designation of the 16 *Klebsiella* genomes in an extended context of 92 genomes from the different *Klebsiella* species. The generated tree annotated with ST (accessible also at https://itol.embl.de/tree/157996425446811617297924) shows that the 16 *Klebsiella* genomes clustered according to the Kraken2 identified taxa. The labels of the 16 *Klebsiella* genomes are colored in red.

SUPPLEMENTARY FIGURE 3 Phylogeny of *Klebsiella* isolates in the present study showing the distribution of MLST profiles. Neighbour-joining based phylogenetic tree of the 13 genomes of *Kp*SC species complex compared based on the loci assigned by the cgMLST analysis in BIGSdb-Pasteur. The tree is visualized on iTOL and available at https://itol.embl.de/tree/1579964253460351617320309.

SUPPLEMENTARY FIGURE 4 Comparative analysis of plasmid carried by *Klebsiella pneumoniae* strains. The figure displays the comparison of the *K. pneumoniae* strains plasmid **(A)** Pkp1-19, (Accession number: CP012884.1) and **(B)** *Klebsiella pneumoniae* strain *bla*_NDM-1_ plasmid p2 (Accession number CP009115.1). In both panels, the outer ring shows the CDS (coding genes) of each plasmid with plasmid replicons. The other rings display the BLAST results of when the genome of *K. pneumoniae* strains MEZKP30, MEZKP33, MEZKP34, MEZKP38, MEZKP45, MEZKP48, MEZKP50, MEZKP186, and MEZKP192 respectively are compared with each of the plasmids. The hits (shaded) regions show regions of homology.
