# Supplementary figures and images for: Phenotypic and genome-based characterization of Klebsiella species from different One Health sources in South Africa reveals the presence of multidrug-resistant isolates
Source: Front Microbiol. 2026 May 29;17:1752622. doi: 10.3389/fmicb.2026.1752622 (PMC13261358; doi:10.3389/fmicb.2026.1752622)

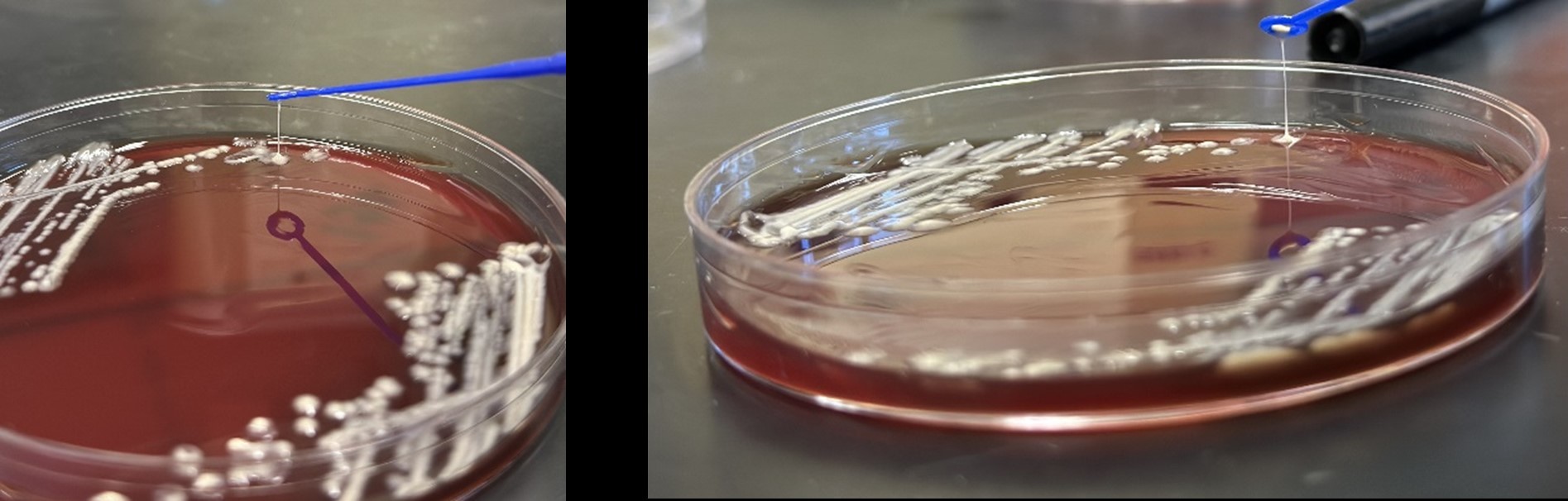

Supplement: Supplementary file 1 [file Data_Sheet_1.zip › Supplementary Figure 1.jpg]

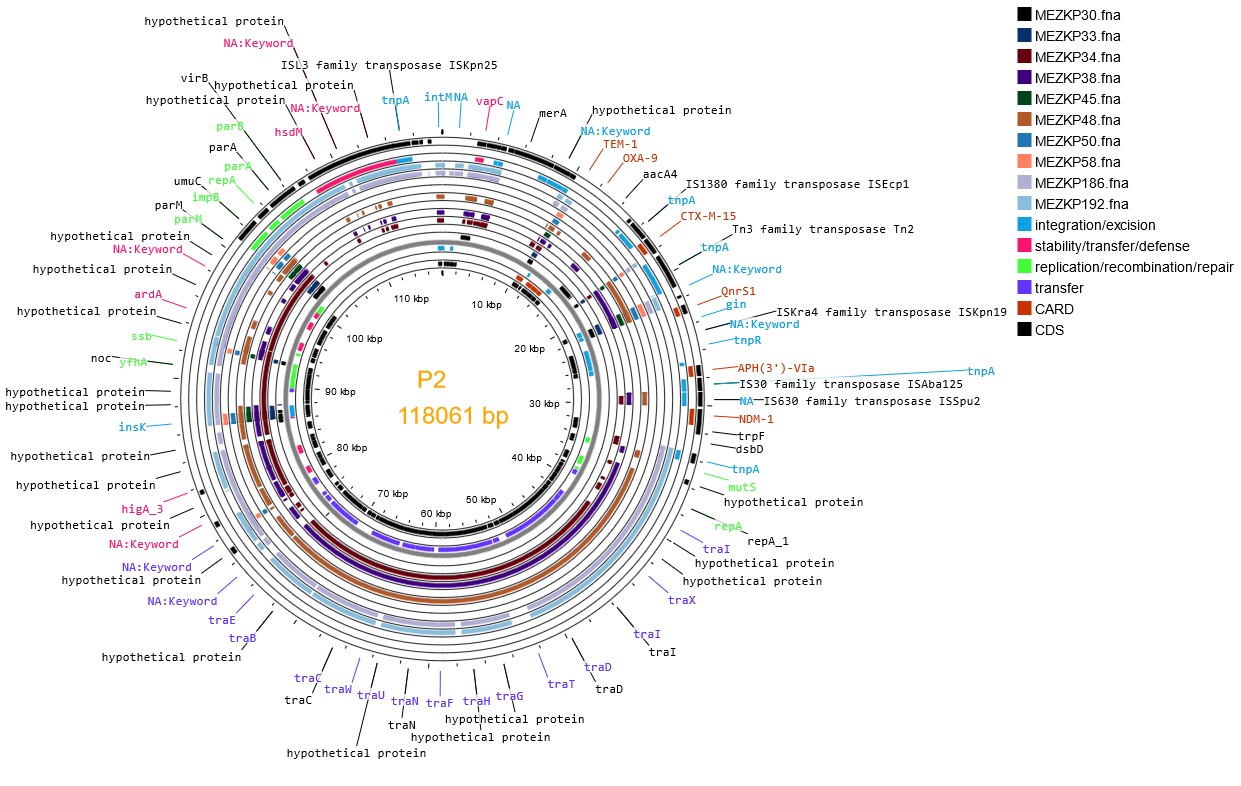

Supplement: Supplementary file 1 [file Data_Sheet_1.zip › Supplementary Figure 4B.jpg]

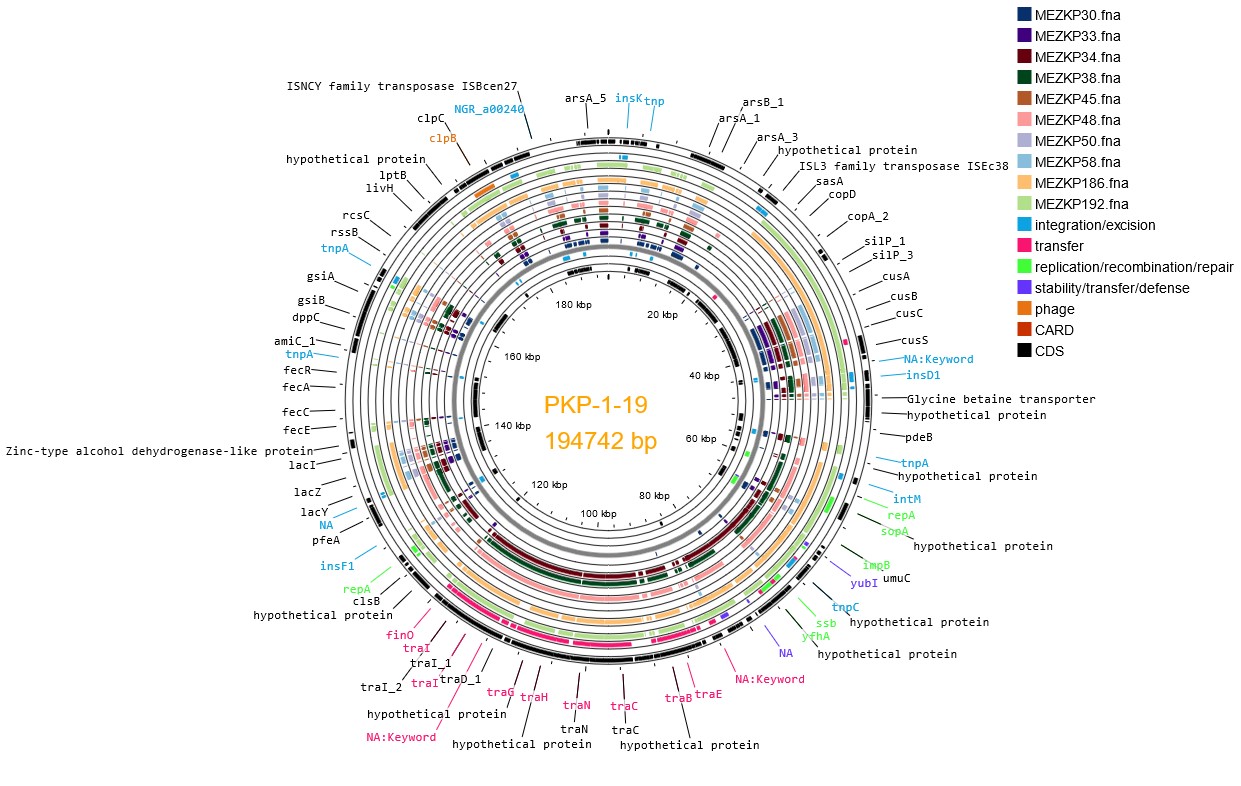

Supplement: Supplementary file 1 [file Data_Sheet_1.zip › Supplementary Figure 4A.jpg]

Tree scale: 0.01

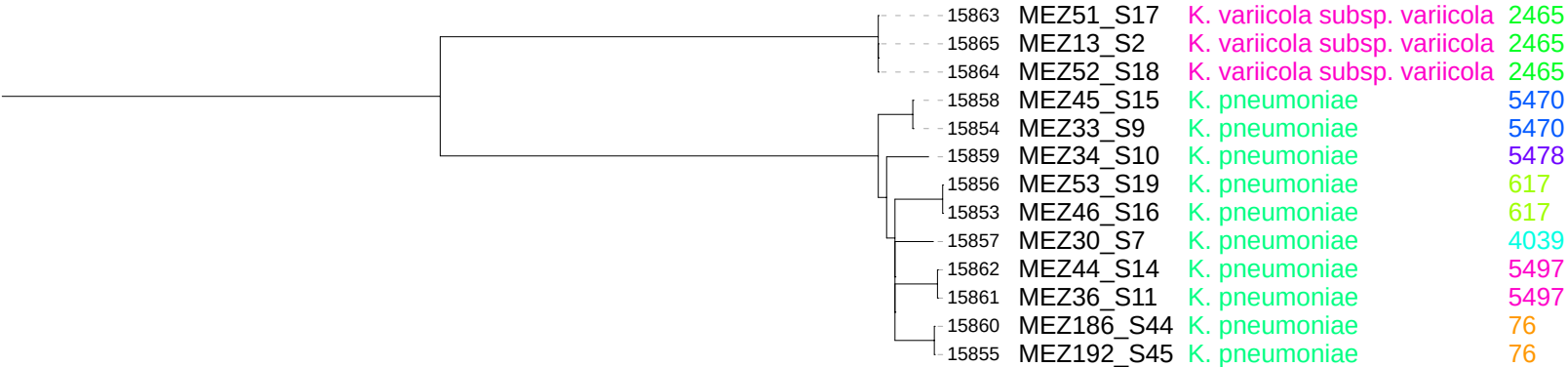

Supplement: Supplementary file 1 [file Data_Sheet_1.zip › Supplementary Figure 3.pdf]

Tree scale: 0.1

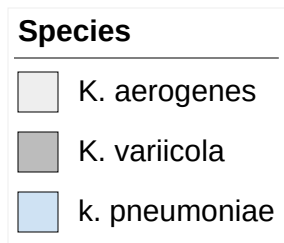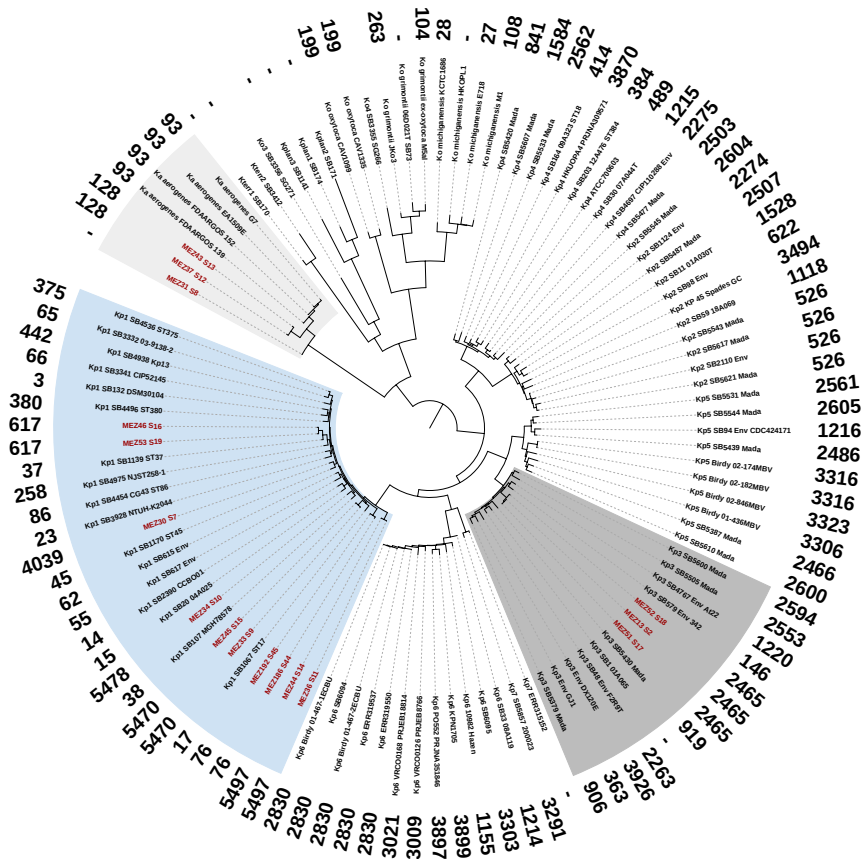

Supplement: Supplementary file 1 [file Data_Sheet_1.zip › Supplementary Figure 2.pdf]
